# Distinct Contributions of the Peroxisome-Mitochondria Fission Machinery During Sexual Development of the Fungus *Podospora anserina*

**DOI:** 10.3389/fmicb.2020.00640

**Published:** 2020-04-15

**Authors:** Raful Navarro-Espíndola, Harumi Takano-Rojas, Fernando Suaste-Olmos, Leonardo Peraza-Reyes

**Affiliations:** Departamento de Bioquímica y Biología Estructural, Instituto de Fisiología Celular, Universidad Nacional Autónoma de México, Mexico City, Mexico

**Keywords:** mitochondria, peroxisome, dynamin-related protein, organelle inheritance, organelle fission, sexual development

## Abstract

Mitochondria and peroxisomes are organelles whose activity is intimately associated and that play fundamental roles in development. In the model fungus *Podospora anserina*, peroxisomes and mitochondria are required for different stages of sexual development, and evidence indicates that their activity in this process is interrelated. Additionally, sexual development involves precise regulation of peroxisome assembly and dynamics. Peroxisomes and mitochondria share the proteins mediating their division. The dynamin-related protein Dnm1 (Drp1) along with its membrane receptors, like Fis1, drives this process. Here we demonstrate that peroxisome and mitochondrial fission in *P. anserina* depends on FIS1 and DNM1. We show that FIS1 and DNM1 elimination affects the dynamics of both organelles throughout sexual development in a developmental stage-dependent manner. Moreover, we discovered that the segregation of peroxisomes, but not mitochondria, is affected upon elimination of FIS1 or DNM1 during the division of somatic hyphae and at two central stages of sexual development—the differentiation of meiocytes (asci) and of meiotic-derived spores (ascospores). Furthermore, we found that FIS1 and DNM1 elimination results in delayed karyogamy and defective ascospore differentiation. Our findings reveal that sexual development relies on complex remodeling of peroxisomes and mitochondria, which is driven by their common fission machinery.

## Introduction

Eukaryotic cell development relies on the regulation of the activity and dynamics of organelles, which involves precise modulation of their composition, abundance, and morphology. Cell development also relies on positional regulation mechanisms that control the distribution of organelles within cells, establishing their appropriate localization at their sites of action, and regulating their distribution and partitioning during cell division and differentiation. Moreover, dynamic interactions and crosstalk between organelles play important roles in the orchestration of their activity during development. Peroxisomes and mitochondria are ubiquitous organelles whose activity in the cell is intimately associated (for review, Fransen et al., 2017). These organelles have long been known for their cooperative function in cell metabolism—including the fatty acid beta-oxidation pathway and the glyoxylate cycle (Kunze et al., 2006; Wanders et al., 2015), and both have a prominent role in reactive oxygen species (ROS) homeostasis and redox regulation (Lismont et al., 2015). Moreover, both organelles can act as signaling platforms that integrate complex signaling pathways (Dixit et al., 2010; Horner et al., 2011). In addition, the processes that regulate peroxisome and mitochondria formation and dynamics—including the transcriptional regulation of their biogenesis (Bagattin et al., 2010), their division (Schrader et al., 2016; Kraus and Ryan, 2017) and their removal from cells (Mao et al., 2014; Zhang et al., 2015; Qi et al., 2016; Lee et al., 2017; Cho et al., 2018; Marcassa et al., 2018)—are interrelated and share key components (for review, Mohanty and McBride, 2013; Fransen et al., 2017). Furthermore, mitochondria contribute to peroxisome biogenesis by providing vesicles that produce peroxisome precursors upon fusion with endoplasmic reticulum (ER)-derived vesicles (Sugiura et al., 2017).

Peroxisomes and mitochondria multiply by division of pre-existing organelles by using common fission machinery. An essential component of this process is the dynamin related-protein Dnm1 (Drp1 in mammals), which assembles into spirals around organelles and undergoes a conformational change upon GTP binding and hydrolysis that facilitates organelle constriction and fission (Bleazard et al., 1999; Sesaki and Jensen, 1999; Smirnova et al., 2001; Koch et al., 2003; Ingerman et al., 2005; Kuravi et al., 2006; Mears et al., 2011). Mitochondrial constriction by Dnm1/Drp1 is preceded by the establishment of ER–mitochondria contacts, which mark the division site and promote an initial actin dynamics-dependent constriction (Friedman et al., 2011; Korobova et al., 2013; for review Pagliuso et al., 2018). Drp1 has severing ability to perform the membrane scission of both mitochondria and peroxisomes (Kamerkar et al., 2018); however, in mammals the dynamin Dyn2 has been proposed to contribute to the final scission of mitochondria (Lee et al., 2016), although this contribution remains controversial (Fonseca et al., 2019). In addition, a second dynamin-like protein—Vps1p—can drive peroxisome fission in the yeast *Saccharomyces cerevisiae* (Hoepfner et al., 2001; Motley and Hettema, 2007). The membrane receptor Mff mediates Drp1 recruitment to mitochondria (along with the chordate-specific Mid49 and Mid51) and peroxisomes (Gandre-Babbe and van der Bliek, 2008; Kraus and Ryan, 2017) in mammals, whereas Dnm1 recruitment in yeasts is mediated by Fis1 along with the paralogous adaptors Mdv1 and Caf4 (Mozdy et al., 2000; Tieu and Nunnari, 2000; Tieu et al., 2002; Griffin et al., 2005; Motley et al., 2008; Nagotu et al., 2008). In mammalian cells, Fis1 also localizes to peroxisomes and mitochondria, and its elimination affects the division of both organelles; however, the precise function of this protein remains elusive (Koch et al., 2005; Schrader et al., 2016; Kraus and Ryan, 2017).

Peroxisome and mitochondrial fission plays different roles in their dynamic regulation, including their distribution and proliferation (Smirnova et al., 1998; Li et al., 2004; Schrader et al., 2016), their inheritance during cell division (Mitra, 2013; Horbay and Bilyy, 2016; Kanfer and Kornmann, 2016), and their elimination by selective autophagy (pexophagy and mitophagy, respectively) (Twig et al., 2008; Manivannan et al., 2013; Mao et al., 2013, 2014; Burman et al., 2017). In addition, mitochondrial fission is linked to metabolism and contributes to cell metabolic adaptation. For example, enhanced mitochondrial fission correlates with decreased bioenergetic efficiency and ROS production, whereas elongated mitochondria are associated with increased oxidative phosphorylation and ATP synthesis, and are believed to more efficiently produce and distribute energy (Liesa and Shirihai, 2013; Mishra and Chan, 2016). Moreover, mitochondrial division is also coupled to mitochondrial DNA (mtDNA) synthesis and dynamics, and accurate mtDNA distribution and transmission depends on this process (Ishihara et al., 2015; Lewis et al., 2016).

As highlighted by human degenerative disorders involving shared peroxisome and mitochondrial dysfunction (Schrader et al., 2015; Fransen et al., 2017), the crosstalk between these organelles can importantly impact developmental processes. However, there is still much to know about the peroxisome-mitochondria interplay during development. Peroxisomes and mitochondria play critical roles during sexual development of the model fungus *Podospora anserina* (Supplementary Figure 1 illustrates *P. anserina* sexual development). In this mycelial ascomycete, alterations in mitochondrial biogenesis due to defective activity of the mitochondrial inner membrane insertase OXA1 (Sellem et al., 2005), or in the respiratory chain due to deficiencies in respiratory complexes I, III, or IV (Dufour et al., 2000; Lorin et al., 2001; Sellem et al., 2007; Maas et al., 2009, 2010) impair sexual development. On the other hand, this process involves precise modulation of peroxisome dynamics and biogenesis (Peraza-Reyes et al., 2011; Takano-Rojas et al., 2016). In addition, different stages of sexual development—including karyogamy, meiotic induction and progression—require specific peroxisome biogenesis factors (peroxins) (Berteaux-Lecellier et al., 1995; Bonnet et al., 2006; Peraza-Reyes et al., 2008, 2011; Suaste-Olmos et al., 2018). Moreover, sexual development also relies on a regulatory system that prevents meiosis progression in absence of mitochondrial citrate synthase, which was identified by its genetic interactions with peroxisomal assembly *PEX2* gene (Ruprich-Robert et al., 2002b), and specific peroxisome assembly mutants exhibit defects in mitochondrial morphology, which are consistent with altered mitochondrial fission (Bonnet et al., 2006). These observations suggest that the function of peroxisomes and mitochondria during sexual development is interrelated, and involves an active crosstalk that influences their dynamics. Here, we analyzed the contribution of peroxisome and mitochondrial dynamics during *P. anserina* sexual development by studying the function of the peroxisome-mitochondrial fission machinery. Previously, in *P. anserina* it was shown that DNM1 elimination extends the life span of the fungus and reduces the germination rate of the meiosis-derived spores (ascospores) (Scheckhuber et al., 2007). However, whether sexual development was affected by DNM1 elimination was not detailed addressed. Here we show that DNM1 and FIS1 play important roles in the regulation of peroxisome and mitochondrial dynamics throughout sexual development, and that the function of these proteins is required for sexual development progression.

## Materials and Methods

### Strains and Culture Conditions

The *P. anserina* strains used in this research are derived from the “S” wild-type. All analyzed strains were homokaryotic (Supplementary Table 1). Standard growth media consisted on M2 minimal medium containing 1.1% dextrin. When required, dextrin was replaced by 0.05% oleic acid (plus 0.2% TWEEN 40 used as emulsifier) as sole carbon source. For ascospore germination G medium supplemented with 0.5% yeast extract was used, and protoplasts were regenerated on RG medium. M2 or RG media were supplemented with hygromycin B (30 or 75 μg mL-1 for constructs derived from pBC-Hygro or pUCHygro, respectively), phleomycin (40 μg mL-1), nourseothricin (40 μg mL-1) or geneticin (G418 sulfate, 100 μg ml-1) when required. Media composition and methods for *P. anserina* can be consulted at http://podospora.i2bc.paris-saclay.fr.

### Nucleic Acid Isolation, Transformation and Plasmids

*P. anserina* genomic DNA purification and transformation were performed according to (Coppin-Raynal et al., 1989). *Streptomyces noursei nat1* gene was obtained from plasmid pAPI509, a derivative from pAPI508 (El-Khoury et al., 2008). *Escherichia coli hph* gene was obtained from pBC-Hygro (Silar, 1995), *P. anserina GPD* (glyceraldehyde-3-phosphate dehydrogenase) gene promoter sequence from pPable (Coppin and Debuchy, 2000), and the mCherry-HygR and GFP-HygR cassettes from plasmids pUC-Cherry and pUC-GFP, respectively (Suaste-Olmos et al., 2018). Oligonucleotide primers used in this research are shown on Supplementary Table 2.

### Gene Sequences

*FIS1 (Pa_3_3970), DNM1 (Pa_1_12670), ATP9-7* (*Pa_7_20*), and *IDH1 (Pa_1_5850)* sequences were obtained from the *P. anserina* genome sequence (Espagne et al., 2008). *DNM1* (Scheckhuber et al., 2007) and *ATP9-7* (Dequard-Chablat et al., 2011) have been previously reported. The predicted protein sequences of these genes are available in the GenBank database under accession numbers CDP26907.1, CDP23627.1, CDP30037.1, and CDP22916.1, respectively.

### Gene Deletions

Mutant strains deleted for *FIS1* and *DNM1* genes were generated by replacing their corresponding ORFs by a selectable marker by homologous recombination. *FIS1* was replaced by *nat1* gene, and *DNM1* by *hph* gene. The gene replacement constructs were generated by Double-joint PCR (Kuwayama et al., 2002) (see Supplementary Table 2 for the oligonucleotide primer sequences). *FIS1* replacement construct consisted on the fusion of: (i) 604 bp of *FIS1* ORF 5′ flanking sequence (amplified by PCR using primers *Fis1-5F* and *Fis1-5R*), (ii) *nat1* gene from pAPI509 (amplified with primers *Fis-NourF* and *Fis-NourR*), and (iii) 743 bp of *FIS1* ORF 3′ flanking sequence (amplified with primers *Fis1-3F* and *Fis1-3R*). *DNM1* replacement construct consisted on the fusion of: (i) 732 bp of *DNM1* ORF 5′ flanking sequence (amplified with primers *Dnm1-5F* and *Dnm1-5R*), (ii) *hph* gene from pBC-Hygro (amplified with primers *Dnm-hphF* and *Dnm-hphR*), and (iii) 610 bp of *DNM1* ORF 3′ flanking sequence (amplified with primers *Dnm1-3F* and *Dnm1-3R*). The final purified fusion PCR products were used to transform protoplasts of a Δ*ku70* strain, and the obtained transformants were crossed to the wild-type strain. Purified Δ*fis1* and Δ*dnm1* homokaryotic strains (issued from uninucleate ascospores, see Supplementary Figure 1) of both mating types (*mat*+ and *mat-*) and on the *KU70*^+^ genetic background were recovered from the progeny of these crosses. Correct gene replacements were verified by PCR analyses (Supplementary Figure 2).

### Gene Complementation Analyses

For the gene complementation assays, protoplasts of the Δ*fis1* and Δ*dnm1* mutants were co-transformed with the pSM334_Genticin vector and a DNA sequence encompassing *FIS1* or *DNM1* wild-type gene, respectively (in a 1:3 molar ratio). These DNA molecules were obtained by PCR using genomic DNA as template and primers Fis1-5F/Fis1-3R or Dnm1-5F/Dnm1-3R, respectively. Δ*dnm1* cells used for transformation had a *FOX2::GFP* allele (see below), allowing to directly analyze peroxisome arrangement in different recovered transformants. Mitochondrial arrangement in these transformants was inspected using MitoTracker Red. For *FIS1*, the Δ*fis1* strain used for transformation possessed both the *FOX2::GFP* allele and a MTS-mCherry-encoding gene (see below). For the analysis of peroxisome and mitochondrial dynamics, nine Δ*dnm1* and two Δ*fis1* geneticin-resistant transformants were randomly selected and analyzed. Six out of nine Δ*dnm1* and all two Δ*fis1* geneticin-resistant transformants showed a restoration of peroxisome and mitochondrial fission and dynamics. For the analysis of sexual development, a Δ*dnm1* and a Δ*fis1* complemented strain were analyzed. For this analysis we took advantage of the recessive nature of Δ*dnm1* and Δ*fis1* sexual development phenotype, and, thus, inspected the restoration of asci formation in perithecia issued from Δ*dnm1* × Δ*dnm1 DNM1*^+^*(EC)* and Δ*fis1* × Δ*fis1 FIS1*^+^*(EC)* heterozygous crosses. For each strain, 300 asci issued from 3 biological replicates (*n* = 100 per experiment) were analyzed.

### Tagging of IDH1 and FOX2

FOX2 and IDH1 were tagged by fusing the 3′ end of their corresponding ORFs to the coding sequence of GFP and mCherry, respectively, at their respective loci. For this, a cassette consisting on the fluorescent-protein coding sequence followed by *hph* gene (GFP-Hyg^R^ or mCherry-Hyg^R^ cassettes, respectively, Suaste-Olmos et al., 2018) was integrated into each respective locus by homologous recombination. IDH1 tagging construct (*IDH1*::mCherry-Hyg^R^::*IDH1-3*′*UTR*) was generated by fusion PCR and consisted on the last 722 bp (excluding the stop codon) of *IDH1* ORF 3′ end (amplified with primers *idh1-F* and *lkt-idh1*), fused in frame to the mCherry-Hyg^R^ cassette from pUC-mCherry (amplified with primers *idh-lkt* and *idh-hph*), and followed by 750 bp of DNA downstream *IDH1* stop codon (amplified with primers *idh1-3F* and *idh1-3R*). FOX2 tagging construct (*FOX2*::GFP-Hyg^R^::*FOX2-3*′*UTR*) consisted on the fusion of: (i) the last 680 bp (excluding the stop codon) of *FOX2* ORF 3′ end (amplified with primers *fox2-F* and *lkt-fox2*), (ii) the GFP-Hyg^R^ cassette from pUC-GFP (amplified with primers *fox-lkt* and *fox-hph*), and (iii) 685 bp of DNA downstream *FOX2* stop codon (amplified with primers *fox2-3F* and *fox2-3R*). The latter construct was cloned into pGEM-T Easy Vector (Promega, Madison, WI, USA), following the provider instructions, yielding plasmid pFS01. Next, the *IDH1*::mCherry-Hyg^R^::*IDH1-3'UTR* PCR construct and the *FOX2*::GFP-Hyg^R^::*FOX2-3*′*UTR* cassette obtained as a *Not*I fragment from plasmid pFS01 were gel-purified and used to transform protoplasts of a Δ*ku70* strain. Randomly selected Hyg^R^ transformants were crossed to the wild type, and the Hyg^R^ marker was recovered in the *KU70*^+^ genetic background. For each gene (*FOX2::GFP* and *IDH1::mCherry*), homokaryotic strains of both mating types issued from uninucleate ascospores were recovered. Plasmid pFS01 was verified by sequencing, and *FOX2* and *IDH1* tagging were verified by PCR analyses and by sequencing.

### Construction of Mitochondria-Targeted mCherry Protein

Fusion PCR was used to generate a DNA construct consisting on mCherry coding sequence (PCR-amplified with primers *mts-gfp-F* and *gfp-R*) fused in frame to the sequence predictably encoding the mitochondrial-targeting signal of *P. anserina* ATP9-7 (Dequard-Chablat et al., 2011) (amplified with primers *mts-F* and *mts-R*) preceded by the minimal promoter sequence (0.35 Kb) of *P. anserina GPD* gene (amplified with *pgpd-F* and *pgpd-R*). This construct (g*pd(p)::ATP9-7::mCherry*) was cloned into pGEM-T Easy Vector, and then obtained as an *EcoR*I restriction fragment to be subcloned into the corresponding site of phleomycin resistance-conferring plasmid pPable. The resulting plasmid (pFS03) was used to transform wild-type cells and phelomycin-resistant transformants were randomly recovered. Three of these strains were backcrossed three times to the wild type. pFS03 *ATP9-7::mCherry* gene was verified by sequencing.

### Genetics, Sexual Reproduction and Vegetative Growth Analyses

The Δ*fis1* and Δ*dnm1* strains expressing fluorescently tagged proteins, as well as the strains simultaneously expressing different fluorescent proteins were constructed by genetic crosses. All the generated recombinant genotypes were obtained in homokaryotic strains (see Supplementary Table 1). Because *DNM1* and *FIS1* deletion affected ascospore formation in homozygous crosses, all Δ*fis1* and Δ*dnm1* generated strains were issued from crosses that were heterozygous for their respective loci. The analysis of sexual development was performed on sexual crosses between homokaryotic strains of opposite mating type. Sexual crosses were performed by growing the two pertinent strains in opposite sides of a M2 plate for 3 days in constant light. After this time, the strains were fertilized by pouring and dispersing 2 mL of sterile water over the surface of the mycelia. Since both strains produce male gametes (spermatia) and female organs (protoperithecia) but fertilization can only be attained if they differ in their mating type, this results in the reciprocal fertilization of the two strains. To inspect sexual development of the gene deletion mutants as female partners in heterozygous crosses, we examined the sexual cells produced within the perithecia formed by the mycelium of the pertinent mutant fertilized with wild-type spermatia of opposite mating type. The reciprocal analysis was performed to inspect the contribution of a mutant as male partner. Mycelial growth was measured by determining the radius of mycelial colonies every 24 h for 4 days. The growth rate was defined as the slope of the linear part of the corresponding growth curve. For each strain, three biological replicates each with triplicates were performed. To avoid alterations in the mycelial growth or in the fertility of the analyzed strains due to storage or senescence of the strains, the mycelial explants used to inoculate the colonies of all these experiments were always issued from young growing cultures (at incubation distances ≤3 cm from the point of inoculation of the ascospore yielding the respective strain).

### Cytology

Sexual cycle cells were fixed in 7.4% paraformaldehyde and processed for fluorescence microscopy as previously described (Thompson-Coffe and Zickler, 1994). Peroxisomes were visualized using mCherry- (Suaste-Olmos et al., 2018) or GFP-tagged (above) versions of *P. anserina* FOX2 expressed from its endogenous locus, or GFP fused to the C-terminal peroxisome targeting signal (PTS1) tripeptide SKL (GFP-PTS1) (Ruprich-Robert et al., 2002a). Mitochondria were stained by MitoTracker Red CMXRos (Molecular Probes, Eugene, OR) (0.5 μM), or were visualized using MTS-mCherry (see above), or with a mCherry-tagged version of *P. anserina* IDH1 expressed from its endogenous locus (see above). Nuclei and mtDNA were stained with DAPI (Molecular Probes) (0.5 μg mL^−1^). For live-cell microscopy, hyphae from entire *P. anserina* colonies grown for 24 h at 27°C on M2 agarose beds were imaged as described before (Suaste-Olmos et al., 2018). To avoid differences in the physiology of the analyzed hyphae due to storage or aging of the strains, the mycelial explants used to inoculate the analyzed colonies were always issued from young growing cultures (at incubation distances ≤3 cm from the point of inoculation of the ascospore yielding the strain). Observations were systematically performed for at least two independent homokaryotic strains (issued from independent ascospores) and on at least three independent colonies. The analysis of peroxisome abundance in hyphal ramifications was done on raw confocal micrographs of the mid-plane of growing leading-hypha ramifications. For each strain, 20–25 hyphal ramifications issued from at least 4 biological replicates (5 hyphal ramifications/replicate) were analyzed. For each ramification, a region of interest (ROI) was drawn around each branch of the ramification in the bright field channel and the ROIs were analyzed for integrated density in the corresponding FOX2-GFP channel. The FOX2-GFP fluorescence per cell area was determined for each hyphal branch, and the ratio of fluorescence between the branches was determined. The analyzes of peroxisomes (FOX2-GFP) in asci and mitochondria (IDH1-mCherry/mtDNA) in ascospores was done on confocal z-series maximum-intensity projections (through entire cell volumes at 0.4 μm intervals) of young asci (8–75 μm long) and ascospore (25–55 μm long)-containing asci, respectively, issued from 3 biological replicates (≥30 asci/replicate for asci, ≥15 asci/replicate for ascospores). Peroxisome abundance in ascospores was estimated in maximum-intensity projections of confocal z-series of asci (through entire cell volumes at 0.5 μm intervals). For each strain, ≥45 asci containing young ascospores (25–50 μm long) issued from 3 biological replicates (≥15 asci/replicate) were analyzed. For each ascus, ROIs were drawn around each ascospore in the bright field channel and they were analyzed for integrated density in the corresponding FOX2-GFP channel. The FOX2-GFP fluorescence per cell area of each ascospore was determined, and the ratio of fluorescence between each pair of sister ascospores was calculated. Results were expressed as the maximum ratio of FOX2-GFP fluorescence between the ascospores of an ascus. For the analyses of FOX2- or IDH1-labeled organelles during sexual development, the analyzed sexual cells were issued from crosses homozygous for their corresponding encoding gene.

### Microscopy

Light microscopy was performed on a Nikon Eclipse E600 microscope and images were collected with a cooled Neo Andor sCMOS camera. Confocal microscopy was done on a Zeiss LSM-800 inverted laser scanning confocal microscope, or in the same system equipped with a temperature chamber (at 27°C, for live-cell imaging). In both systems we used a Plan-Apochromat 63x/1.4 oil immersion objective and 405, 488, and 561 nm laser lines, and bright field images were acquired using the Electronically Switchable Illumination and Detection (ESID) module. For time-lapse microscopy, images from all channels were collected simultaneously. For 3D imaging, z-section images were collected at 0.3–0.5 μm intervals through entire cell volumes, except were indicated. Images were processed on ImageJ (NIH, Bethesda, USA) (Schneider et al., 2012) FIJI package (Schindelin et al., 2012) or on ZEN 2012 software (Carl Zeiss, Jena, Germany).

## Results

### FIS1 and DNM1 Elimination Affects Hyphal Growth in *P. anserina*

To investigate the role of the peroxisome-mitochondrial fission machinery in *P. anserina* development we deleted *FIS1* and *DNM1* genes. First, we found that the *P. anserina* mycelial growth rate on standard dextrin-containing medium was reduced upon deleting *FIS1* (0.185 mm/h) or *DNM1* (0.188 mm/h), as compared to the wild type (0.249 mm/h) (Supplementary Figures 3A,B). Then we observed that the Δ*fis1* (0.205 mm/h) and Δ*dnm1* (0.211 mm/h) growth rate on oleic acid-based medium was also reduced, relative to the wild type (0.281 mm/h) (Supplementary Figures 3A,B). The extent of the growth rate reduction of the mutants was similar in both media (25.8% decrease for Δ*fis1* in oleate vs. 27.1%, in dextrin; 24.4 vs. 25% for Δ*dnm1*), suggesting that *FIS1* and *DNM1* deletion does not strongly affect fatty-acid catabolism. These results show that the *P. anserina* hyphal growth is diminished when there are defects in the peroxisome-mitochondrial fission machinery.

### Mitochondrial Division and Arrangement Depend on FIS1 and DNM1

**Figure 1:**
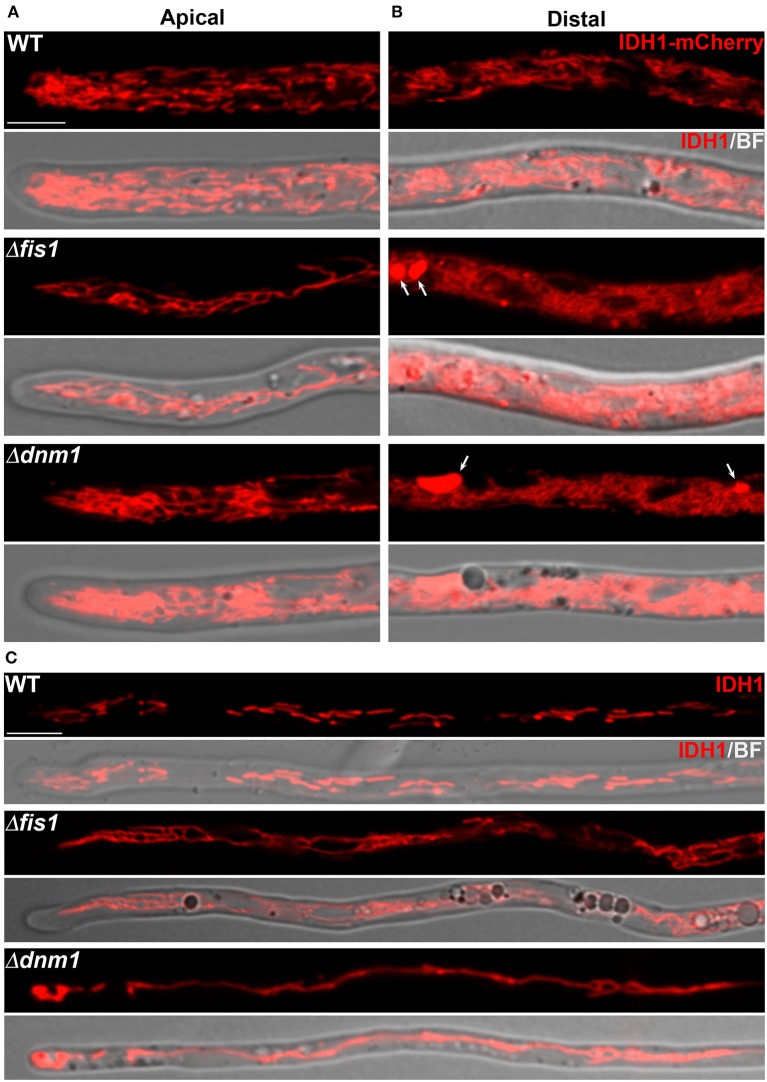
FIS1 and DNM1 are required for mitochondrial division and arrangement. Confocal microscopy analysis of IDH1-mCherry-labeled mitochondria in different hyphal regions of wild type (WT), Δ*dnm1* and Δ*fis1* mycelia. **(A,B)** Mitochondrial morphology in the apical **(A)** and distal (**B**, ~100 μm behind the hyphal tip) regions of leading hyphae. **(C)** Mitochondrial morphology in narrow branch hyphae emerging from distal hyphal regions. BF, bright field. Arrows point to large spherical mitochondria. Scale bar, 5 μm.

**Figure 2:**
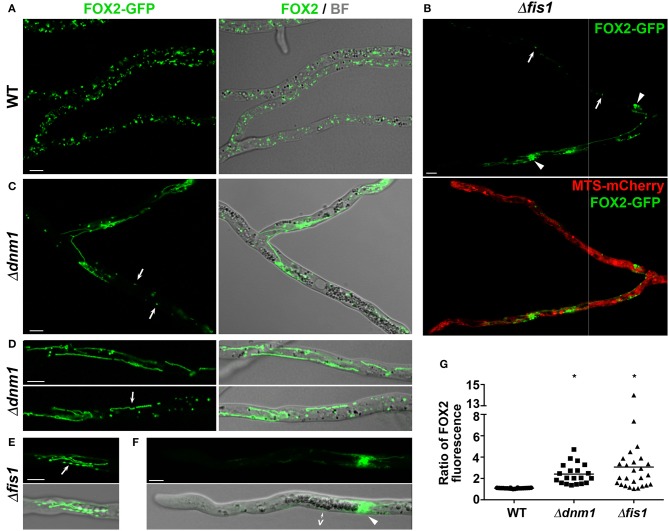
FIS1 and DNM1 are required for peroxisome division and distribution. Peroxisome arrangement in hyphae of WT **(A)**, Δ*fis1* **(B,E,F)**, and Δ*dnm1* **(C,D)** strains expressing FOX2-GFP. In **(B)** peroxisomes are compared to the localization of MTS-mCherry-labeled mitochondria. Arrows in **(B,C)** point to punctate peroxisomes and in **(D,E)** to beads-on-a-string peroxisomal chains. Arrowheads in **(B)** indicate large spherical peroxisomes and in **(F)** clustered peroxisomes next to the vacuolized hyphal region (*v*). BF, bright field. Scale bar, 5 μm. **(G)** Peroxisome distribution between the two branches of hyphal ramifications of WT, Δ*fis1*, and Δ*dnm1* mycelia. Values express the ratio of the FOX2-GFP fluorescence intensity of one hyphal branch relative to the other, and are plotted in a scatter plot; *n* ≥ 20. *P = <0.05 by unpaired Student's *t*-test.

It was previously demonstrated that DNM1 elimination in *P. anserina* results in mitochondrial elongation (Scheckhuber et al., 2007), whereas its overproduction enhances mitochondrial fragmentation (Scheckhuber et al., 2008). However, the requirement of FIS1 for mitochondrial fission, or the participation of both proteins in peroxisome fission in this fungus has not been addressed. First, we analyzed the effect of *FIS1* deletion on mitochondrial dynamics in hyphae by inspecting the localization of a C-terminally mCherry-tagged version of the putative mitochondrial NAD(+)-dependent isocitrate dehydrogenase IDH1, whose gene was tagged at its chromosomal locus. IDH1-mCherry stained a dynamic network of tubular structures in the wild type that is consistent with mitochondrial localization (Figures 1A,B, Supplementary Movie 1). Importantly, we did not observe defects in the growth or development of the strains expressing IDH1-mCherry (shown for mycelia in Supplementary Figure 3C), suggesting that the tagging did not affect IDH1 function. Consistent with participation in mitochondrial fission, we observed that *FIS1* deletion resulted in elongation of IDH1-mCherry-labeled mitochondria (Figure 1A, Supplementary Movie 2). This morphology was similar to the previously described for Δ*dnm1* Mitotracker Red-labeled mitochondria (Scheckhuber et al., 2007). However, we also observed the formation of extensive networks of interwoven mitochondria (Figure 1B), as well as large spherical mitochondria (Figure 1B, arrows), which were not previously described in *P. anserina* Δ*dnm1* mutants. Therefore, to corroborate the mitochondrial nature of these structures we visualized mitochondria using a second mitochondrial marker. We generated strains expressing an ectopically-encoded mitochondrion-targeted mCherry protein (MTS-mCherry) (Supplementary Figure 4A, Supplementary Movie 3), which possesses the mitochondrial-targeting signal of *P. anserina* F_0_ATP synthase subunit *c* (ATP9-7, Dequard-Chablat et al., 2011). Mitochondrial arrangement of Δ*fis1* hypha as visualized with MTS-mCherry (Supplementary Figure 4A, Supplementary Movie 4, see also Figure 2B) was very similar to the one observed with IDH1-mCherry, supporting the mitochondrial nature of the labeled organelles. In addition, we analyzed mitochondria by staining cells with MitoTracker red (Supplementary Figure 5) and found that the large spherical Δ*fis1* mitochondria were not clearly labeled with this dye, suggesting that they have reduced membrane potential. Finally, we also found that Δ*fis1* cells contained chains of mitochondria arranged as beads-on-a-string, from which no individual mitochondria were detached. The arrangement of these mitochondrial chains was relatively uniform, but constrictions defining large mitochondrial bulges were also formed (Supplementary Movie 5). This finding is consistent with a function of FIS1 in the later steps of mitochondrial fission and not in the formation of the initial constrictions leading mitochondrial division.

Next, we observed that the mitochondrial arrangement of Δ*dnm1* hyphae expressing IDH1-mCherry was very similar to that of Δ*fis1* (Figure 1, Supplementary Movie 6), and that the Δ*dnm1* large spherical mitochondria were also not clearly stained with MitoTracker (Supplementary Figure 5C). Interestingly, Δ*fis1* and Δ*dnm1* mitochondrial arrangement exhibited a differential distribution throughout mycelia. In *P. anserina* leading hyphae, the growing apical region extending ≈50 μm behind the tip is followed by a region where large spherical vacuoles accumulate. In Δ*fis1* and Δ*dnm1*, the leading hyphae apical segments contained elongated mitochondria, whereas large spherical mitochondria mainly accumulated behind the vacuolar region (Figures 1A,B). For example, in Δ*dnm1* hyphae—where the vacuolar region is located 45.7 ± 9.6 μm behind the hyphal tip—the large mitochondria appeared, in average, 63.8 ± 13.3 μm behind the tip (*n* = 18). Furthermore, the networks of interwoven mitochondria were more profuse and exhibited a tighter packaging toward the hyphal distal parts (Figures 1A,B). In addition, we observed that Δ*fis1* and Δ*dnm1* narrow hyphae branching from subapical/distal regions of mycelia contained mostly highly elongated mitochondria (Figure 1C). Importantly, Δ*fis1* and Δ*dnm1* mitochondrial arrangement was restored upon ectopically reintroducing a wild-type copy of *FIS1* or *DNM1* gene, respectively, into the corresponding mutant (Supplementary Figures 4A,B), corroborating that the described phenotypes are caused by the generated gene deletions.

### FIS1 and DNM1 Are Required for Peroxisome Division

Then we analyzed the effect of *FIS1* and *DNM1* deletion on peroxisome dynamics by studying the localization of C-terminally GFP- and/or mCherry-tagged versions of the endogenous fatty-acid β-oxidation enzyme FOX2. FOX2 is a peroxisomal protein that lacks canonical peroxisome-targeting signals (PTS), but whose import into peroxisomes is dependent on the import receptor PEX5. We have shown that FOX2 peroxisome targeting is not affected by the C-terminal tagging (Suaste-Olmos et al., 2018). In addition, for Δ*fis1* we corroborated our observations using an ectopically-encoded GFP possessing the consensus peroxisome-targeting signal 1 (GFP-PTS1). We found that elimination of FIS1 or DNM1 resulted in extensive peroxisome elongation (Figures 2A–E, see also Supplementary Figure 6), showing that both proteins are actually required for peroxisome fission. However, Δ*fis1* and Δ*dnm1* hyphae also contained punctate (Figures 2B,C, arrows) and large spherical (arrowheads) peroxisomes. In addition, and similar to mitochondria, we also observed that elongated peroxisomes frequently adopted a beads-on-a-string arrangement (Figures 2D,E, arrows), from which no peroxisomes were separated (Supplementary Movie 7). These peroxisomal chains were highly dynamic and the constrictions defining their arrangement were repeatedly redistributed over time, produced large transient bulges or eventually disappeared, reverting peroxisomes to a tubular shape. This shows that FIS1 are DNM1 mediate the later steps of peroxisome fission, but are not required for the initial organelle constriction.

Different metabolic and environmental cues regulate peroxisome dynamics in *P. anserina*, including oleic-acid utilization, which induce peroxisome proliferation, and low temperature exposure, which promotes peroxisome elongation (Takano-Rojas et al., 2016). We analyzed the effect of *FIS1* and *DNM1* deletion on peroxisome dynamics under these stimuli and we observed that the peroxisome elongation induced by cold (4°C for 12 h**)** was exacerbated in Δ*fis1* or Δ*dnm1* hyphae; however, these cells still contained punctate peroxisomes (Supplementary Figure 6A). Also, we found that, like for the WT, the peroxisomal amount of Δ*fis1* and Δ*dnm1* hyphae increases upon growth on oleic acid, as compared to dextrin (Supplementary Figures 6A,B). However, in contrast to the WT punctate peroxisomes, most Δ*fis1* and Δ*dnm1* peroxisomes were highly elongated or displayed a beads-on-a-string arrangement. These results show that DNM1 and FIS1 are not required for the proliferation of peroxisomes *per se*, but for the scission producing their individualization.

### The Cell Distribution of Peroxisomes Depends on FIS1 and DNM1

Next we observed that peroxisome distribution in Δ*fis1* and Δ*dnm1* mycelia was heterogeneous. We found that a number of hyphal segments contained low numbers of peroxisomes, more notably in the apical region of the mycelium leading hyphae (Figures 2B,F, Supplementary Movies 4, 7, 8). These segments were frequently preceded by clusters of elongated interwoven peroxisomes, whose displacements were seemingly obstructed by other organelles, most notably by the vacuoles at the subapical region (Figure 2F, see also the lower hyphae in Supplementary Movies 4, 8), or that were retained at the sites of hyphal ramifications (Figures 2B,C, Supplementary Movie 8, see also Supplementary Figure 5C). This defect resulted in asymmetric peroxisome segregation during the ramification of hyphae (e.g., Figure 2B, Supplementary Movie 4). We quantified this defect by determining the ratio of peroxisome FOX2-associated fluorescence (per cell area) between the two branches of leading-hyphae ramifications (Figure 2G), and we observed that in 61.5% of Δ*fis1* and 65% of Δ*dnm1* ramifications (*n* ≥ 20/strain) the peroxisome-associated fluorescence was at least 2-fold higher for one hyphae of the ramification (overall av. ratio 2.41 for Δ*dnm1*, 3.06 for Δ*fis1*). In contrast, the difference in peroxisome-associated fluorescence between the two hyphal branches in WT ramifications was below 15% (av. ratio 1.08, *n* = 20). These findings show that *FIS1* or *DNM1* deletion result in uneven peroxisome segregation during hyphal ramification. Re-introducing a wild-type *FIS1* or *DNM1* allele into the respective mutant restored both peroxisome division and distribution (Supplementary Figures 4A,B), confirming that both phenotypes are due to *FIS1* and *DNM1* deletions. Importantly, we did not observe defective mitochondrial partitioning in Δ*fis1* and Δ*dnm1* hyphal ramifications (Figure 2B).

We also inspected whether FIS1 or DNM1 elimination affected peroxisome or mitochondrial inheritance during the formation of spermatia (microconidia), which are small uninucleate single cells that act as male gametes and that originate from aerial branches arising from vegetative hyphae. We found that Δ*fis1* and Δ*dnm1* spermatia contained FOX2-labeled peroxisomes that were elongated (Supplementary Figure 7A, arrows) or displayed a beads-on-a-string arrangement (Supplementary Figure 7A, arrowheads). However, the majority of these cells possessed only punctate peroxisomes. In addition, we observed a slight decrease in the number of spermatia containing peroxisomes in Δ*dnm1* (89.3%) or Δ*fis1* (94.3%) mutants, as compared to the wild type (100%) (Supplementary Figure 7B). These observations suggest a minor role for FIS1 and DNM1 in peroxisome inheritance during spermatia formation. Nonetheless, since peroxisomes could conceivably be produced *de novo* within spermatia, the extent of the peroxisome inheritance deficiency during Δ*dnm1* and Δ*fis1* spermatia formation could be underestimated. WT spermatia typically contained IDH1-mCherry-stained shortly elongated mitochondria or in limited networks. We observed increased mitochondrial elongation in Δ*fis1* and Δ*dnm1* spermatia (Supplementary Figure 7C, arrows), as well as large spherical mitochondria (Supplementary Figure 7C, arrowhead). Still, short mitochondria were abundant in these cells. Notably, we found that, 95.4% of Δ*fis1* and 97% of Δ*dnm1* spermatia contained mitochondria, compared to 98% in the WT (Supplementary Figure 7D), indicating that FIS1 and DNM1 are dispensable for mitochondria inheritance into spermatia.

### Peroxisome Division, Distribution and Segregation During Sexual Development Depend on FIS1 and DNM1

**Figure 3:**
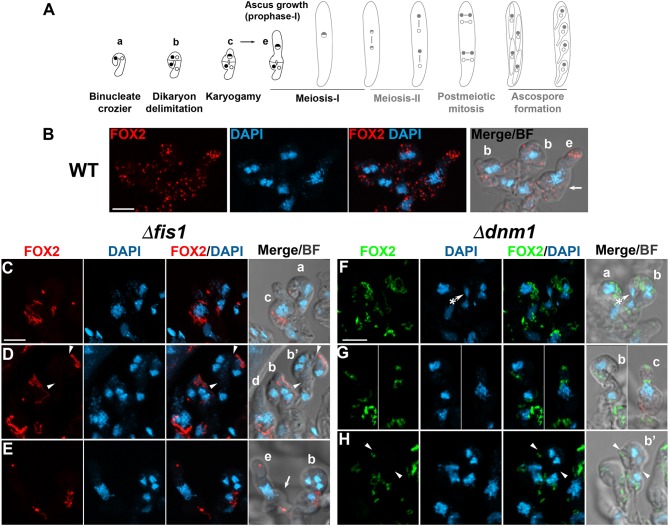
FIS1 and DNM1 elimination affects peroxisome division and segregation during dikaryotic cell compartmentalization. **(A)** *P. anserina* sexual development from the dikaryotic stage to ascospore formation (from left to right, for further details consult Supplementary Figure 1), the developmental stages analyzed in this figure are depicted in black. Analysis of FOX2-labeled peroxisomes at the dikaryotic stage of WT **(B)**, Δ*fis1* **(C–E)**, and Δ*dnm1* **(F–H)** homozygous crosses. Small letters indicate successive developmental stages: (a) young binucleated croziers. (b) Dikaryotic cell formation [arrowheads in **(D,H)** indicate the septa delimiting the dikaryotic cell; note the lateral cell nucleus—asterisk in **(F)** migrating into the basal cell to produce a new dikaryotic cell]. (c) Karyogamy. (d,e) show asci at successive stages of meiotic prophase-I [arrows in **(B,E)** indicate the reminiscent initial crozier cell]. (b′) indicates croziers whose dikaryotic cell lacks peroxisomes. DNA was stained with DAPI. Fluorescence images show maximum-intensity projections of z series through the entire cells. Bright field (BF) merged images show single plane micrographs. Scale bar, 5 μm.

**Figure 4:**
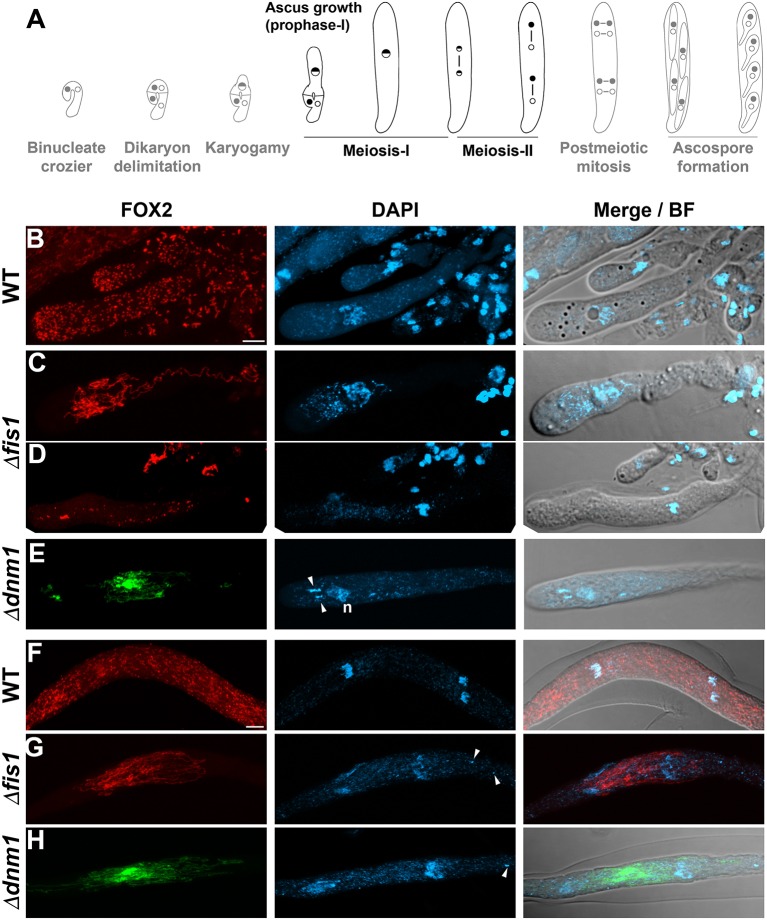
FIS1 and DNM1 elimination affects peroxisome division and distribution during meiocyte differentiation. **(A)** *P. anserina* sexual development from the dikaryotic stage to ascospore formation (from left to right, for further details consult Supplementary Figure 1), the developmental stages analyzed in this figure are depicted in black. **(B–H)** Analysis of FOX2-labeled peroxisomes during meiotic development in WT, Δ*fis1*, and Δ*dnm1* homozygous crosses. **(B–E)** Show the peroxisome arrangement in growing meiotic prophase-I asci, and **(F–H)** in asci at the end of meiosis. Arrowheads point to fragments of non-nuclear genetic material (n, nucleus). Fluorescence images show maximum-intensity projections of z series through the entire cells. Bright field (BF) merged images show single plane micrographs. Scale bar, 5 μm.

**Figure 5:**
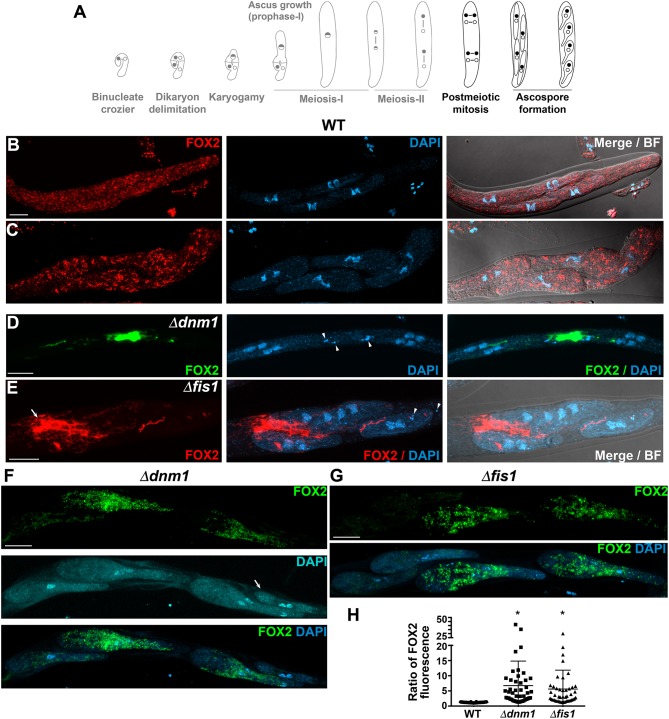
FIS1 and DNM1 elimination affects peroxisome division and inheritance during ascospore differentiation. **(A)** *P. anserina* sexual development from the dikaryotic stage to ascospore formation (from left to right, for details consult Supplementary Figure 1), the developmental stages analyzed in this figure are depicted in black. Analysis of FOX2-labeled peroxisomes during ascospore differentiation in WT **(B,C)**, Δ*dnm1* **(D,F)** and Δ*fis1* **(E,G)** homozygous crosses. **(B,D,E)** Ascospore formation, **(B,E)** show asci with newly formed ascospores and **(D)** at post-meiotic mitosis before ascospore delineation. In **(E)** note the cluster of elongated peroxisomes that fail to be incorporated into ascospores (arrow). **(C,F,G)** Growing ascospores. In **(F,G)** note the uneven partitioning of peroxisomes into ascospores, and in **(F)** the aberrant undifferentiated ascospore (arrow). Arrowheads point to fragments of non-nuclear genetic material. Fluorescence images show maximum-intensity projections of z series through the entire cells. Bright field (BF) merged images show single plane micrographs. Scale bar, 10 μm. **(H)** Peroxisome distribution between the sister ascospores of asci issued from WT, Δ*fis1*, and Δ*dnm1* homozygous crosses. Values express the ratio of the FOX2-GFP fluorescence intensity between the two ascospores showing the largest difference in peroxisome abundance per ascus, and are plotted in a scatter plot; *n* ≥ 45. *P = <0.05 by unpaired Student's *t*-test.

*P. anserina* is an heterothallic ascomycete that reproduces exclusively sexually in a process involving the formation of multicellular fructifications called perithecia. Strains of different mating type start sexual reproduction by forming spermatia and female gametangia (ascogonia), which cross-fertilize. Ascogonia are wrapped by a protective coat and upon fertilization develop into perithecia. The ascogonial cells present within perithecia then produce specialized hook-shaped cells called croziers, in which dikaryotic compartmentalization takes place. Subsequently, the crozier dikaryotic cell undergoes karyogamy, enters meiosis and differentiates into an ascus (the meiocyte), where meiosis occurs. The four nuclear products of meiosis then divide mitotically, and the eight resulting nuclei are packed two-by-two into four ascospores, which grow and differentiate inside the original mother ascus (Supplementary Figure 1, Figures 3A, 4A, 5A). By inspecting the localization of FOX2, we found that FIS1 and DNM1 elimination disturbs peroxisome division, distribution and segregation during sexual development (Figures 3–5). These results are described in the next sections.

### *FIS1* and *DNM1* Deletion Affects Peroxisome Division and Segregation During Meiocyte Formation

Young binucleated croziers divide by synchronized mitoses and septa formation producing three cells: a binucleated cell possessing opposite mating-type nuclei (the dikaryotic cell) flanked by two uninucleated cells (Supplementary Figure 1, Figure 3A). The dikaryotic cell differentiates into an ascus, while the uninucleated cells fuse to produce a new dikaryotic crozier. Wild-type croziers possess mostly punctate FOX2-labeled peroxisomes (Figure 3B, cells *b*). In contrast, most Δ*fis1* or Δ*dnm1* croziers contained elongated peroxisomes, which were often asymmetrically distributed (Figures 3C,D, cells *b*-*c*; and Figure 3F). In addition, we found that some Δ*fis1* and Δ*dnm1* croziers contained few peroxisomes (Figures 3C,E, cells *a*-*b;* and Figure 3G). Actually, we found that 18.4% of Δ*fis1* croziers (*n* = 76) and 23.2% of Δ*dnm1* (*n* = 56) lacked FOX2-labeled peroxisomes in their dikaryotic cells (Figures 3D,H, cells *b*′), compared to 1.8% in the wild type (*n* = 55). Also, some mutant croziers even entirely lacked peroxisomes (6.3% for Δ*fis1, n* = 127; 4.3% for Δ*dnm1, n* = 70), a condition not observed in the wild type (*n* = 83).

The crozier dikaryotic cell undergoes karyogamy and differentiates into an ascus, which elongates from around 5 to more than 150 μm along meiosis prophase-I (Supplementary Figure 1, Figure 4A). During this process, wild-type peroxisomes exhibit a punctate pattern (Figure 4B, see also cell *e* in Figure 3B for an early ascus); in contrast, most Δ*fis1* and Δ*dnm1* meiotic prophase-I asci possessed networks of elongated peroxisomes (Figures 4C,E, Supplementary Movie 9). However, we also observed a number of early meiotic prophase-I asci (i.e., 13.1% for Δ*fis1, n* = 107; 18% for Δ*dnm1, n* = 100), which contained few mostly punctate peroxisomes (shown for Δ*fis1 in* Figure 4D, see also cell *e* in Figure 3E for an early ascus). These asci were not observed in the wild type (*n* = 90). Yeast cells lacking peroxisomes due to defective peroxisome segregation are able to produce peroxisomes *de novo* (Motley and Hettema, 2007; Wroblewska and van der Klei, 2019). Actually, the peroxisomes produced *de novo* in cells deficient for peroxisome fission emerge as multiple small punctate peroxisomes (Motley et al., 2015). Therefore, the latter asci could be derived from crozier dikaryotic cells lacking peroxisomes, and their punctate peroxisomes might represent *de novo* produced peroxisomes. These observations are consistent with a defect in peroxisome segregation during meiocyte differentiation when FIS1 and DNM1 are missing.

### FIS1 and DNM1 Are Required for Peroxisome Partitioning During Ascospore Formation

*DNM1* and *FIS1* deletion resulted in extensive peroxisome elongation in fully elongated asci (shown for asci ending meiosis in Figures 4F–H). These cells contained networks of highly elongated interwoven peroxisomes that frequently formed densely packed clusters with asymmetric distribution. This distribution remained until post-meiotic mitoses (shown for Δ*dnm1* in Figure 5D), and during ascospore formation resulted in uneven peroxisome partitioning into nascent ascospores (shown for Δ*fis1* in Figure 5E, compare to Figure 5B), and in large networks of elongated peroxisomes that failed to be incorporated into these cells (arrow in Figure 5E). Consistent with defective peroxisome segregation, the sister ascospores of Δ*fis1* or Δ*dnm1* asci often contained uneven amounts of peroxisomes (Figures 5F,G). We quantify this defect by determining the maximum ratio of peroxisome FOX2-GFP-associated fluorescence (per cell area) between the sister ascospores of an ascus (i.e., the ratio of the FOX2-GFP fluorescence intensity between the two ascospores showing the largest difference in peroxisome abundance per ascus, Figure 5H). We observed that 72% of Δ*dnm1* (*n* = 46) and 80% of Δ*fis1* (*n* = 45) asci contained at least two sister ascospores differing by, at minimum, 2-fold in their peroxisomal amount. Overall, the average maximum ratio of FOX2 fluorescence between the sister ascospores of an ascus was 5.6 ± 6.23 (median 3.08) for Δ*dnm1*, and 6.73 ± 8 (median 4.3) for Δ*fis1*, compared to 1.2 ± 0.1 (median 1.19, *n* = 46) in the wild type. Importantly, we also observed that Δ*fis1* and Δ*dnm1* produced small, as well as very small aberrant undifferentiated ascospores (see below). Of note, in 82.6% of Δ*dnm1* and 75.6% of Δ*fis1* asci, the ascospore containing the lowest amount of FOX2-labeled peroxisomes (per cell area) corresponded to the smallest ascospore of the ascus. Furthermore, the aberrant undifferentiated ascospores contained very limited numbers of peroxisomes (Figure 5F, arrow), as compared to their sister ascospores (i.e., av. maximum ratio of FOX2 fluorescence against undifferentiated ascospores for Δ*dnm1*: 10.9 ± 9.4, median 6.2, *n* = 13; for Δ*fis1*, 9.98 ± 9.8, median 7.1, *n* = 24). These results indicate that peroxisome segregation during ascospore differentiation depends on FIS1 and DNM1, and shows a correlation between the peroxisome segregation and ascospore formation defects. Interestingly, in addition to their uneven segregation, peroxisomes of Δ*fis1* and Δ*dnm1* growing ascospores were mostly rounded—ranging from punctate to globular—and also exhibited an asymmetric distribution within ascospores (Figures 5F,G). This arrangement contrasted with the mostly elongated of other cell types.

### Elimination of FIS1 and DNM1 Differently Affects Mitochondrial Arrangement at Different Stages of Sexual Development

**Figure 6:**
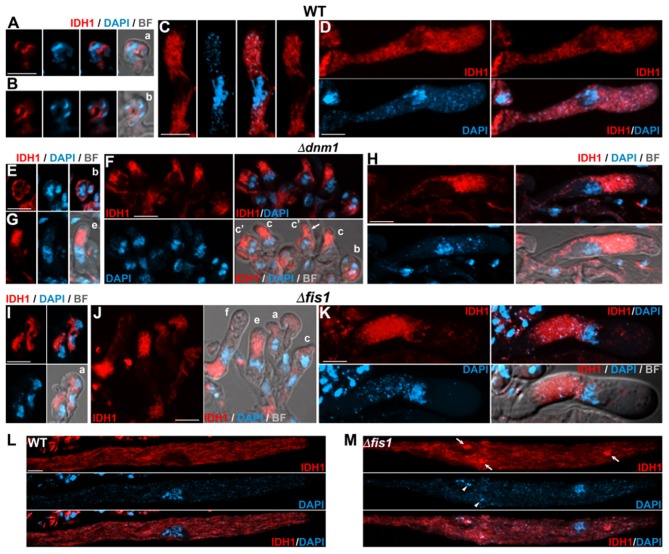
FIS1 and DNM1 elimination affects mitochondrial dynamics during meiocyte differentiation. Analysis of IDH1-mCherry-labeled mitochondria in early sexual development stages from dikaryotic croziers to meiocyte formation in WT **(A–D)**, Δ*dnm1* **(E–H)**, and Δ*fis1* **(I–K)** homozygous crosses. Small letters indicate successive developmental stages as in Figure 3. (*c*′) indicates croziers whose dikaryotic cell has started differentiating an ascus (arrow) but has not undergone karyogamy. Mitochondrial arrangement in WT **(L)** and Δ*fis1* **(M)** late meiotic prophase-I asci. Arrows indicate mitochondrial clumps and arrowheads fragments of non-nuclear genetic material. For clarity, images **(L,M)** show z-series maximum-intensity projections of the ascus mid-plane (1 μm in depth), and **(A–C)** (right panel) and **(D)** (upper right) show single plane images; the remaining fluorescence images show z-projections through the entire cells. Bright field (BF) merged images show single plane micrographs. Scale bar, 5 μm.

Next we studied the effect of *FIS1* and *DNM1* deletion on mitochondrial arrangement during sexual development. In wild-type croziers, IDH1-mCherry stained elongated or shortly elongated discrete mitochondria (Figures 6A,B), whereas during asci formation it labeled larger mitochondrial networks (Figure 6C), which displayed a more reticulate pattern during ascus growth (Figure 6D). We observed elongated defined mitochondria in Δ*fis1* and Δ*dnm1* croziers (Figure 6E), but most of these cells possessed reticulate (Figure 6J, cells *a* and *c*) or densely packed (Figures 6F,I) mitochondrial clusters. Early Δ*fis1* and Δ*dnm1* asci also possessed densely packed clusters of mitochondria (Figures 6G,J cells *e, f*), which were more profuse following ascus growth (Figures 6H,K). These clusters were asymmetrically distributed, next to the nucleus, and their pattern was reminiscent of that of mitochondria in distal hyphal regions.

**Figure 7:**
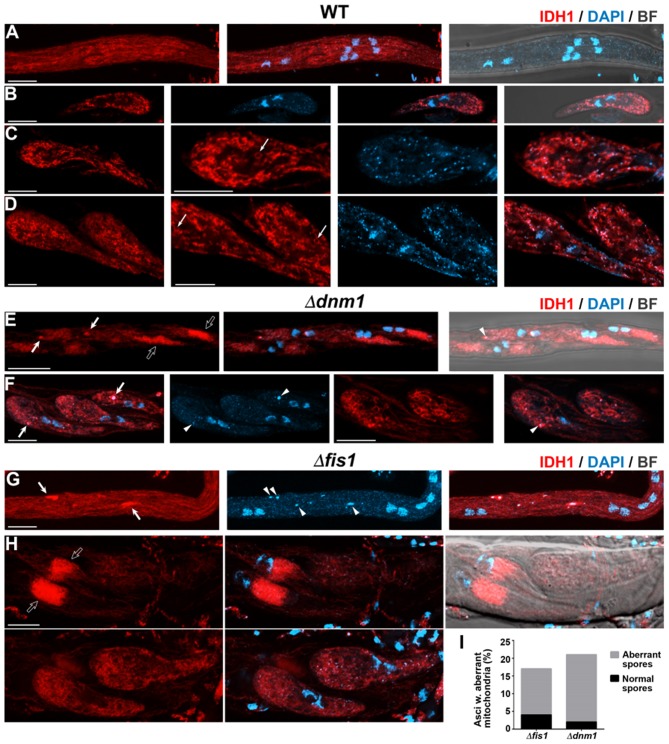
FIS1 and DNM1 elimination affects mitochondrial dynamics during ascospore differentiation. Analysis of IDH1-mCherry-labeled mitochondria during ascospore differentiation of WT **(A–D)**, Δ*dnm1* **(E,F)**, and Δ*fis1* **(G,H)** homozygous crosses. **(A,E,G)** Ascospore formation. **(B–D,F,H)** Growing ascospores; **(B–D)** show progressive stages of ascospore differentiation. For clarity, images in **(B)** show single plane micrographs, and the right panels in **(C,D,F)** show single plane magnifications of the corresponding left panel cells. In **(H)** note that two pairs of spores of the ascus overlap, the upper panels show the maximum-intensity z-projection of the focal planes encompassing the left spores, while the lower panels show the corresponding to the right spores. The remaining images show z-projections through the entire cells. In **(C,D)** arrows indicate ring-like mitochondria. In **(E–H)** arrows indicate mitochondrial clumps, arrowheads fragments of non-nuclear genetic material and open arrows large aberrant mitochondrial clusters. Scale bar, 5 μm. **(I)** Quantitation of Δ*fis1* and Δ*dnm1* asci containing spores with large aberrant mitochondrial clusters, the occurrence of asci containing aberrant undifferentiated ascospores is indicated, *n* ≥ 45.

Following ascus elongation (Figure 6L) and early during ascospore formation (Figure 7A) WT asci contained abundant elongated mitochondria. This arrangement was similar in Δ*fis1* and Δ*dnm1* asci (shown for Δ*fis1* in Figures 6M, 7G), except that the mitochondrial networks were more heterogeneous and contained large mitochondrial clumps (arrows). Following ascospore formation, wild-type mitochondria exhibited a more fragmented pattern (shown for progressive ascospore differentiation stages in Figures 7B–D). Interestingly, in these cells some mitochondria adopted an spherical ring-like structure (thin arrows). Δ*fis1* and Δ*dnm1* ascospores contained networks of fused elongated mitochondria (Figures 7F,H, lower panels), which frequently also contained mitochondrial clumps (Figures 7E–G, arrows). In addition, we found that a number of ascospores harbored mitochondria consisting of large tightly packaged clusters (shown for two developmental stages in Figures 7E,H, upper panels). These mitochondria displayed a perinuclear distribution and exhibited a similar arrangement to that of growing asci. We observed that 21.3% of Δ*dnm1* (*n* = 47) and 17.8% of Δ*fis1* (*n* = 45) asci contained at least one ascospore with this abnormal mitochondrial arrangement (Figure 7I). Moreover, these mitochondria were only observed in small mutant ascospores, which in most cases (10/11 for Δ*dnm1*, 8/10 for Δ*fis1*) corresponded to aberrant undifferentiated spores. Overall, we found that 43% (*n* = 23) and 47% (*n* = 19) of Δ*dnm1* and Δ*fis1* aberrant ascospores, respectively, contained these tightly packed mitochondrial clusters. Importantly, we did not observe defects in mitochondria segregation along Δ*fis1* or Δ*dnm1* sexual development.

### Elimination of FIS1 and DNM1 Affects the Distribution of mtDNA

We found that Δ*fis1* and Δ*dnm1* asci contained fragments of DAPI-stained genetic material, which were larger and brighter than regular mitochondrial nucleoids but that were not associated to nuclear DNA (e.g., arrowheads in Figures 4, 5). Double labeling experiments demonstrated that these DNA fragments co-occurred with IDH1-mCherry-stained mitochondria, mostly at mitochondrial clumps (Figures 6M, 7E–G), suggesting that they represent clustered nucleoids. We quantified the presence of clustered nucleoids in ascospores and found that they were present in 28.6% (*n* = 189) and 28.7% (*n* = 178) of Δ*dnm1* and Δ*fis1* ascospores, respectively. These observations indicate that the alteration of mitochondria dynamics due to FIS1 or DNM1 elimination has repercussions on mtDNA distribution. Nonetheless, we did not observe compromised mtDNA segregation in Δ*fis1* or Δ*dnm1* ascospores.

### Karyogamy Is Delayed in Absence of FIS1 or DNM1

**Figure 8:**
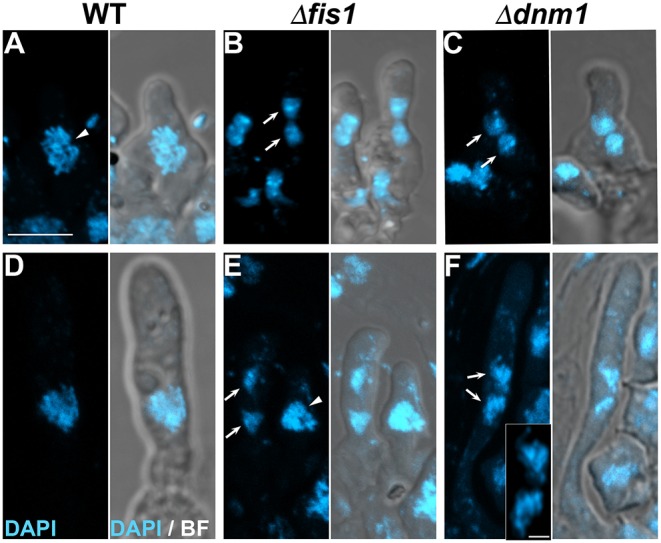
Karyogamy is delayed in absence of FIS1 or DNM1. Nuclear distribution in early differentiating (1–5 μm in length) **(A–C)** and young (5–20 μm long) **(D–F)** asci issued from WT, Δ*fis1*, and Δ*dnm1* homozygous crosses. Arrows point to unfused nuclei and arrowheads to post-karyokamy nuclei. Inset in **(F)** show a high-resolution magnification of the unfused nuclei. Scale bar, 5 μm; inset scale bar, 1 μm.

Next, we studied the effect of FIS1 and DNM1 elimination in sexual development. We have shown that peroxisomes are required for karyogamy and meiotic development initiation in *P. anserina* (Berteaux-Lecellier et al., 1995; Peraza-Reyes et al., 2008, 2011; Suaste-Olmos et al., 2018); therefore, we tested whether FIS1 and DNM1 elimination affected these processes. As shown before, Δ*fis1* and Δ*dnm1* are able to produce ascospores in homozygous crosses, implying that FIS1 and DNM1 are not strictly required for karyogamy of meiosis initiation. However, we also observed a ≈24 h delay in Δ*fis1* and Δ*dnm1* sexual development. Then, we analyzed the nuclear distribution in early stages of meiocyte formation and we observed a number of early differentiating asci containing unfused nuclei at stages were the wild type has normally undergone karyogamy. We determined the number of croziers whose dikaryotic cell has started differentiating an ascus but still contains unfused nuclei. We observed that 17.5% of Δ*fis1* (*n* = 80) and 24.7% of Δ*dnm1* croziers (*n* = 73) with emerging asci ranging from 1 to 5 μm in length possessed nuclei that have not suffered karyogamy, compared to 11.6% in the wild type (*n* = 60), where in most cases the emerging asci was 1–2 μm in length (Figures 8A–C). Unfused nuclei in Δ*fis1* and Δ*dnm1* asci longer than 5 μm were infrequent, i.e., 6.5% for Δ*fis1* (*n* = 62) and 8.2% for Δ*dnm1* (*n* = 61) in asci ranging from 5 to 20 μm; still, they were not observed in the wild type (Figures 8D–F). These observations indicate that elimination of DNM1 or FIS1 results in a moderate delay in karyogamy.

### FIS1 and DNM1 Are Required for Ascospore Differentiation

**Figure 9:**
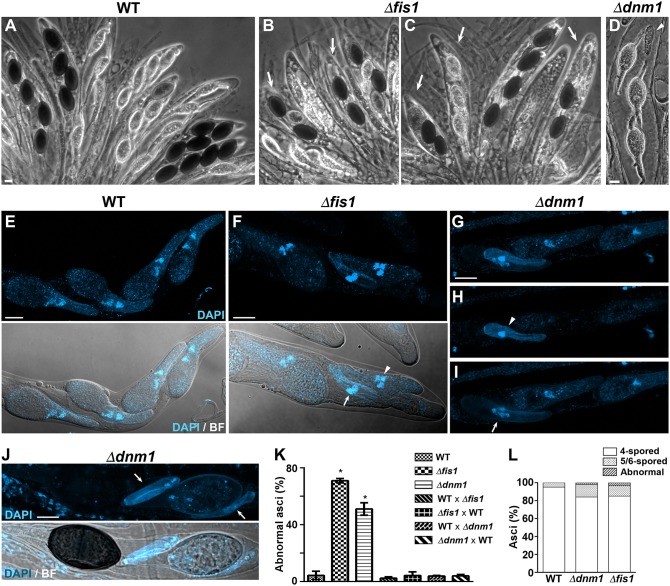
FIS1 and DNM1 are required for ascopsore differentiation. **(A–D)** Asci issued from WT, Δ*fis1*, and Δ*dnm1* homozygous crosses. Arrows point to abnormal asci containing ascospores in irregular numbers (note that only 2–3 ascospores are detectable, in contrast to the four ascospores of WT asci) or of uneven sizes (compare to the WT ascospores or even size). The arrowhead shows an aberrant undifferentiated ascospore. **(E–J)** Nuclear distribution in WT **(E)**, Δ*fis1* **(F)**, and Δ*dnm1* **(G–J)** ascospores. In **(F)** the arrow shows a small aberrant binucleated ascospore and the arrowhead a small ascospore with abnormal number of nuclei. In **(G)** note that two spores are superimposed, **(H,I)** show the maximum-intensity z-projections of the focal planes encompassing each individual spore, respectively (arrow, small binucleated ascospore; arrowhead, small aberrant ascospore). **(J)** Shows an ascus concluding maturation. Note that the small aberrant ascospores (arrows) remained undifferentiated when their sister ascospores were fully differentiated. Scale bar, 10 μm. **(K)** Quantitation of abnormal asci in the indicated sexual crosses. Δ*fis1* and Δ*dnm1* correspond to homozygous crosses. WT × Δ*fis1* (or Δ*dnm1*) correspond to heterozygous crosses in which the WT acted as female partner, Δ*fis1* × WT corresponds to the reciprocal cross (i.e., Δ*fis1* acting as female partner) (*n* ≥ 300 in three independent experiments. *P <0.05 by unpaired Student's *t*-test, relative to WT). **(L)** Quantitation of the ascospore nuclear distribution in Δ*fis1* and Δ*dnm1* asci. Asci containing ascospore nuclear distributions different from four binucleated ascospores (4-spored), three binucleated and two uninucleated ascospores (5-spored) or two binucleated and four uninucleated ascospores (6-spored) were considered abnormal (*n* ≥ 150).

We discovered that Δ*dnm1* and Δ*fis1* asci frequently contained ascospores of uneven sizes and/or in irregular numbers (Figures 9A–D). We observed that Δ*dnm1* and Δ*fis1* formed small ascospores (Figures 9B,C, see also Figure 9I), as well as very small aberrant ascospores, which did not differentiate head and tail cells (Figure 9D, arrowhead; see also Figures 5F, 9F,H) and remained undifferentiated throughout the entire ascus development (Figure 9J). These defects were observed in around 50% of Δ*dnm1* and 70% of Δ*fis1* asci issued from homozygous crosses, but not in heterozygous crosses of either Δ*fis1* or Δ*dnm1* to the wild type, indicating a recessive phenotype (Figure 9K). Also, we observed that Δ*fis1* and Δ*dnm1* ascospore formation was significantly restored by genetically complementing the mutants with ectopic wild-type *FIS1* or *DNM1* alleles, respectively (Supplementary Figure 4C), corroborating that these defects were caused by the *FIS1* and *DNM1* deletions.

In *P. anserina*, defects in the size and number of ascospores produced in an ascus can reflect defective nuclear progression during meiotic and/or post-meiotic development. In wild-type strains, ≈98% of asci form four binucleated ascospores; however, in a small percentage (≈2%), one of these ascospores is replaced by two small uninucleated ascospores, resulting in asci containing three large binuclated ascospores and two small uninucleated (Supplementary Figure 1). This results from altered orientation of one of the post-meiotic mitosis spindles (Zickler et al., 1995). Eventually, asci containing six ascospores (two binucleated and four uninucleated) can also be produced. We analyzed nuclear distribution in Δ*dnm1* and Δ*fis1* asci and observed some ascospores containing uneven numbers of nuclei (Figure 9E–G); however, the number of asci with altered ascospore nuclear distribution was very low (2 and 3.4%, respectively, Figure 9L), indicating that the defect in Δ*dnm1* and Δ*fis1* ascospore formation was not caused by a defective meiotic/post-meiotic nuclear progression. Nonetheless, we observed that the number of 5- and 6-spored asci was increased in Δ*dnm1* (14%) and Δ*fis1* (11.6%), as compared to the wild type (5%) (Figure 9L). These observations suggest a minor impact of FIS1 and DNM1 elimination on meiotic/post-meiotic development in *P. anserina*.

## Discussion

Research on sexual development in fungi has provided relevant information on the cellular and molecular basis of fundamental developmental processes, like karyogamy, meiosis and gametogenesis. Research in the mycelial fungus *P. anserina* has shown that sexual development involves precise regulation of peroxisome biogenesis and dynamics, and that this organelle is required for the establishment and progression of key events of sexual development, including karyogamy and meiotic development (Bonnet et al., 2006; Peraza-Reyes et al., 2011; Takano-Rojas et al., 2016; Suaste-Olmos et al., 2018). In addition, specific mitochondrial functions are also required for progression through sexual development, and evidence suggests that the activity of mitochondria and peroxisomes during this process is interrelated (Ruprich-Robert et al., 2002b; Bonnet et al., 2006). Here we present evidence that mitochondria dynamics is also subject to a developmental regulation during the sexual cycle, and that the dynamics of both organelles during this process depends on the conserved fission proteins DNM1 and FIS1. Furthermore, we show that affecting the activity of the peroxisome-mitochondrial fission machinery has an important detrimental effect on sexual development in this fungus.

As for other studied organisms, we found that peroxisomes and mitochondria use common division machinery in *P. anserina*. We showed that FIS1 and DNM1 elimination results in extensive peroxisome and mitochondrial elongation. In addition, absence of these proteins also resulted in constricted chains of mitochondria and peroxisomes, from where no individual organelles were separated. Similar mitochondrial constrictions are spontaneously produced in normal animal cells by a rearrangement of the inner membrane, which is referred to as Constriction of Mitochondrial Inner Compartments (CoMIC). These constrictions serve as priming event for mitochondria division and their formation is enhanced by Drp1 inhibition (Cho et al., 2017). Also, ultrastructural analyses have shown that similar peroxisomal chains produced by DLP1 (Drp1) inhibition in mammalian cells actually constitute constricted undivided peroxisomes (Koch et al., 2004). These observations indicate that the scission of both organelles, and not their initial constriction, actually depend on FIS1 and DNM1 in *P. anserina*.

Nonetheless, we also observed that Δ*fis1* and Δ*dnm1* cells contained numerous punctate peroxisomes, even in conditions of extensive peroxisome elongation. Whether these peroxisomes result from *de novo* peroxisome biogenesis or from additional fission processes remains to be determined. However, the *P. anserina* genome possesses an ortholog of *S. cerevisiae VPS1* gene, suggesting an additional peroxisome fission process. Also, in different Δ*fis1* and Δ*dnm1* cell types we observed fragmented mitochondria. Mitochondria destined to mitophagy can divide in a process that is concurrent to autophagosome formation but independent of Dnm1/Drp1 (Yamashita et al., 2016). Thus, it is possible that the mitochondria fragmentation observed in *P. anserina* cells lacking DNM1 is related to this process. In addition, we also observed that mitochondria were segregated during the formation of different cell types, including ascospores and spermatia (microconidia), when FIS1 or DRP1 were missing. Similarly, Fis1 and Dnm1 are dispensable for mitochondria inheritance into conidia in *Aspergillus fumigatus* (Neubauer et al., 2015). This indicates that *P. anserina* is able to divide mitochondria during the formation of these cells in absence of FIS1 or DNM1. Although evidence for Dnm1/Drp1-independent mitochondria division processes has been obtained (Murakawa et al., 2015; Roy et al., 2016; Yamashita et al., 2016), an alternative mechanism accounting for mitochondria division along these processes is currently unknown. Conceivably, mitochondria (and peroxisomes) could indirectly be cleaved by the cell division machinery driving cell individualization during these processes, as it has previously been proposed for mitochondria division during cytokinesis in *S. cerevisiae dnm1* mutants (Sesaki and Jensen, 1999).

Consistent with the participation of the mitochondria division machinery in mtDNA replication and distribution (for review, Pagliuso et al., 2018), we found that mtDNA distribution was also affected by the elimination of FIS1 or DNM1. In *S. cerevisiae*, the fission proteins Fis1 and Mdv1 are required for mtDNA maintenance; however, Dnm1 seems to be dispensable for this process, suggesting different functions for these proteins in mtDNA maintenance in this yeast (Bradshaw et al., 2012). In *P. anseina*, elimination of either FIS1 or DNM1 resulted in nucleoid clustering predominantly at regions were large bulged mitochondrial were produced. Actually, in mammalian cells, Drp1 inhibition promotes the clustering of nucleoids within hyperfused mitochondria, which results in cristae stacking and formation of large bulb-like mitochondria (Ban-Ishihara et al., 2013).

On the other hand, we found that the elimination of FIS1 and DNM1 produces different defects in the arrangement of peroxisomes and mitochondria; including the formation of large spherical organelles, as well as tightly packaged clusters of mitochondria. Consistent with our observations, large spherical mitochondria are also produced upon *DNM1* or *FIS1* deletion in *A. fumigatus* (Neubauer et al., 2015), and bulb-like mitochondria (Mopert et al., 2009; Otera et al., 2010) as well tight clusters of mitochondrial tubules (Smirnova et al., 1998) are also produced in mammalian cells affected in Drp1 activity. Interestingly, in *P. anserina* these distinct mitochondrial morphologies displayed a differential distribution throughout hyphae. This shows that the cellular context influences the outcome of impaired mitochondrial fission, and indicates that the regulation of mitochondria dynamics is subject to different hypha regional constrains.

In addition, we also discovered that FIS1 and DNM1 elimination affects peroxisome and mitochondrial dynamics throughout all sexual development stages, underscoring a foremost role for the peroxisome-mitochondria fission machinery in regulating the dynamics of both organelles throughout the sexual cycle. Again, we observed that FIS1 and DNM1 elimination differentially affected peroxisome and mitochondrial dynamics at distinct stages of sexual development, implying also different developmental constrains in the modulation of organelle dynamics.

The dynamics of different organelles, including the Golgi complex, vacuoles, peroxisomes, and mitochondria, is regulated during cell division to allow proper organelle segregation between daughter cells (Kanfer and Kornmann, 2016; Knoblach and Rachubinski, 2016; Ayala and Colanzi, 2017). Importantly, the proteins of the peroxisome-mitochondria fission machinery, notably Drp1/Dnm1, play a central role in the regulation of mitochondria partitioning. During mitosis in mammals, interphase cells have interconnected networks of elongated mitochondria, which fragment early at mitosis and then re-fuse following cytokinesis, reforming a mitochondrial network in daughter cells (Taguchi et al., 2007; Mitra et al., 2009). Mitochondrial fission at mitosis is driven by Drp1 (Taguchi et al., 2007), which is regulated at multiple levels by key cell cycle regulators (Taguchi et al., 2007; Zunino et al., 2009; Horn et al., 2011; Kashatus et al., 2011). Actually, failure to induce mitochondrial fission at mitosis results in uneven mitochondrial inheritance during cell division (Taguchi et al., 2007; Kashatus et al., 2011). In turn, mitochondria fission influences cell cycle progression (Mitra, 2013; Lopez-Mejia and Fajas, 2015). In *P. anserina* we found that the distribution of peroxisomes is affected when DNM1 or FIS1 are missing; specially, we discovered that the partitioning of peroxisomes during the division of the leading growing hypha of mycelia is unequal when their fission is defective. Moreover, we found that DNM1 and FIS1 elimination also affects peroxisome segregation at two fundamental sexual differentiation stages—meiocyte and ascospore differentiation. These findings show that in *P. anserina* Dnm1-dependent peroxisome fission is important for correct peroxisome segregation both in somatic cells and in the sexual cycle. Still, we cannot exclude that additional cellular processes—more notably pexophagy—are also affected by FIS1 and DNM1 elimination, and that these deficiencies could contribute to the altered distribution of peroxisomes in these cells. Importantly, we did not observe defective mitochondria segregation in any Δ*dnm1* or Δ*fis1* developmental stage, indicating different constrains for the segregation of organelles of different nature, even when sharing key elements in the regulation of their dynamics.

The actual participation of the fission machinery in regulating peroxisome distribution and segregation remains unknown. However, it is possible that the motility of peroxisomes is hindered by the morphological alterations they suffer when their division is defective. Conceivably, the movement of the clusters of elongated peroxisomes generated upon FIS1 or DNM1 elimination could be physically obstructed in hyphal regions with particular architectural traits—like hyphal branching points or septal pores—or with high intracellular crowding. In filamentous fungi, peroxisomes are transported along microtubules by early endosomes, with whom they establish physical interactions (Guimaraes et al., 2015; Salogiannis et al., 2016). Thus, the dynamics of the interactions between endosomes and peroxisomes, or the correct endosome-mediated association of peroxisomes with microtubule motor proteins, which drive their displacements, could also be affected by the alterations in peroxisome morphology generated when they are unable to divide. Therefore, peroxisome severing by the fission machinery could promote the formation of small peroxisomes, which could be more readily transported along microtubule tracks, and facilitate their defined associations with endosomes and, hence, with the microtubule-based transport machinery. The observation that mitochondria distribution in Δ*dnm1* or Δ*fis1* mutants was not as severely altered as that of peroxisomes suggests different mechanisms involved in the regulation of their distribution. This notion is consistent with the finding that mitochondrial transport in *Ustilago maydis* is not dependent on endosome motility (Guimaraes et al., 2015).

Meiotic development also involves extensive organelle remodeling that ensures proper organelle partitioning during the formation of the cellular products of meiosis. A well-documented example is provided in *S. cerevisiae*, where mitochondria associate with the cell cortex in premeiotic cells through interactions with the plasma membrane and ER, to be detached and redistributed to the perinuclear area at meiosis-II. Mitochondria then remain surrounding each nucleus until ascospore delimitation, when they divide and are evenly distributed into ascospores (Miyakawa et al., 1984; Gorsich and Shaw, 2004; Sawyer et al., 2019). Actually, the correct partitioning of mitochondria during this process depends on the fission proteins, including Dnm1 and Fis1 (Gorsich and Shaw, 2004). In *P. anserina*, mitochondria partitioning during ascospore formation was not notably affected upon FIS1 or DNM1 elimination. Nonetheless, the segregation of peroxisomes during this process was severely compromised, stressing the distinct systems that have evolved in different organisms to regulate their organelle segregation. Importantly, these observations are also consistent with the existence of a more stringent mechanism for mitochondria segregation, which ensures the segregation of this organelle that, in contrast to peroxisomes, cannot be produced *de novo*.

Nevertheless, we observed that mitochondria morphology in Δ*fis1* and Δ*dnm1* ascospores varied, and some spores presented tightly packed mitochondrial clusters that were frequently associated with small ascospores, which could indicate that the activity of these mitochondria is hampered. In mammalian cells, the failure to promote the Drp1-dependent mitochondrial fission at mitosis leads to a loss of mitochondrial ATP production and a decrease in the number of metabolically active cells over time (Kashatus et al., 2011). Furthermore, the asymmetric distribution of old and young mitochondria during cell division of mammalian stemlike cells, which promote stemness in daughter cells inheriting younger mitochondria, is diminished when Drp1 activity is inhibited (Katajisto et al., 2015). Thus, it is tempting to speculate that rather than the quantity; the quality of the inherited mitochondria is compromised in Δ*fis1* and Δ*dnm1*.

Peroxisome inheritance has been shown to play a critical role in the regulation of mitosis and cell differentiation in mammalian epidermal progenitor cells (Asare et al., 2017). Interestingly, in the *P. anserina* Δ*fis1* and Δ*dnm1* mutants, we observed defects at both developmental stages were peroxisome inheritance was affected. In *P. anserina*, peroxisomes are required for dikaryotic cells to undergo karyogamy and differentiate into meiocytes (Berteaux-Lecellier et al., 1995; Peraza-Reyes et al., 2008, 2011; Suaste-Olmos et al., 2018). Our finding that karyogamy is delayed in the Δ*fis1* and Δ*dnm1* mutants is consistent with the observation that a number of dikaryotic cells fail to inherit peroxisomes in these mutants. Still, this defect was moderate and we did not observed complete inability to undergo karyogamy or to form asci, as occurs in peroxisome biogenesis mutants. This could be due to the fact that peroxisomes can be produced *de novo*. Still, the mitochondrial alterations of the dikaryotic cells lacking FIS1 or DNM1 could also contribute to this karyogamy delay.

The developmental process that was more critically affected in absence of FIS1 or DNM1 was the formation of ascospores, the process equivalent to gametogenesis in plants and animals. We found that the differentiation of many Δ*fis1* and Δ*dnm1* ascospores did not progress beyond their initial delineation, and that this defect was not associated to defective meiotic nuclear progression. In addition, we showed that this phenotype was a recessive trait. This indicates that the failure underlying this defect occurs at a very early differentiation stage, following nuclear segregation and before ascospores become autonomous. The actual contribution of the peroxisome-mitochondrial fission machinery to this process remains unclear. However, the various defects produced by *FIS1* and *DNM1* deletion, including defective peroxisome inheritance and severe mitochondrial morphology alterations, suggest a complex implication for these proteins. Moreover, organelle fission through DRP1 also contributes to additional intracellular dynamics processes, notably pexophagy and mitophagy, which provide quality control mechanisms to remove low-functioning organelles and their damaged proteins (Twig et al., 2008; Manivannan et al., 2013; Mao et al., 2013, 2014). Furthermore, Drp1/Dnm1 can exert an important impact on cell fate during development through its influence on cell cycle progression and in key signaling pathways (reviewed in Mitra, 2013; Lopez-Mejia and Fajas, 2015; Noguchi and Kasahara, 2018), and thereby regulate cell differentiation (e.g., Mitra et al., 2012). Therefore, it is possible that ascospore formation in *P. anserina* involves the multifaceted activity of these fission proteins.

## Data Availability Statement

The datasets generated for this study are available on request to the corresponding author.

## Author Contributions

RN-E, HT-R, FS-O, and LP-R designed and performed experiments. LP-R wrote the manuscript and obtained funding. All authors contributed to manuscript revision, read, and approved the submitted version.

### Conflict of Interest

The authors declare that the research was conducted in the absence of any commercial or financial relationships that could be construed as a potential conflict of interest.

## Supplementary Material

Supplementary Movie 1 Mitochondrial dynamics in wild-type hyphae. Growing wild-type hypha expressing IDH1-mCherry (**top**). (**Bottom**) IDH1/bright-field merge. Time scale is in min:sec. Scale bar, 5 μm.

Supplementary Movie 2 Mitochondrial dynamics in Δ*fis1* hyphae. Growing Δ*fis1* hypha expressing IDH1-mCherry (**top**). (**Bottom**) IDH1/bright-field merge. Time scale is in min:sec.

Supplementary Movie 3 Peroxisome and mitochondrial dynamics in wild-type hyphae. Growing wild-type hypha expressing FOX2-GFP (**left**) and MTS-mCherry (**center**). (**Right**) FOX2/MTS- mCherry merge. Time scale is in min:sec.

Supplementary Movie 4 Peroxisome and mitochondrial dynamics in Δ*fis1* hyphae. Growing Δ*fis1* hypha expressing MTS-mCherry (**left**) and FOX2-GFP (**center**). (**Right**) MTS-mCherry/FOX2/bright-field merge. Note the low peroxisome abundance in the upper hyphal branch, and the presence of small punctate peroxisomes. Time scale is in min:sec. Scale bar, 5 μm.

Supplementary Movie 5 Mitochondrial constrictions in Δ*fis1* hyphae. Detail of the dynamics of MTS-mCherry-labeled mitochondria in Δ*fis1* hyphae. The movie shows a magnification of the apical region of the upper hypha shown in Supplementary Movie 4. (**Right**) MTS-mCherry/bright-field merge. Time scale is in min:sec. Scale bar, 5 μm.

Supplementary Movie 6 Mitochondrial dynamics in Δ*dnm1* hyphae. Growing Δ*dnm1* hypha expressing IDH1-mCherry (**top**). (**Bottom**) IDH1/bright-field merge. Note the spontaneous formation of numerous mitochondrial constrictions simultaneously toward the end of the movie. Time scale is in min:sec.

Supplementary Movie 7 Peroxisome dynamics in Δ*dnm1* hyphae. Growing Δ*dnm1* hypha expressing FOX2-GFP (**top**). (**Bottom**) FOX2/bright-field merge. Time scale is in min:sec.

Supplementary Movie 8 Peroxisome dynamics during Δ*fis1* hyphal division. Live-cell imaging of FOX2-GFP-labeled peroxisomes (**left**) in a growing leading-hypha ramification. (**Right**) FOX2/bright-field merge. Time scale is in min:sec. Scale bar, 5 μm.

Supplementary Movie 9 Defects in the morphology of peroxisomes in a Δ*fis1* elongating ascus. 3D reconstruction of FOX2-mCherry-labeled peroxisomes in a Δ*fis1* elongating meiotic profase-I ascus. The rendered reconstruction of peroxisomes (red) is shown by moving along the X-axis, and then the distribution of nuclear and mtDNA (blue) is shown. The 2D projection of the 3D volume is shown in Figure 4C.

Supplementary Figure 1 *Podospora anserina* sexual development from the dikaryotic stage to ascospore formation (from left to right). *P. anserina* is an heterothallic ascomycete possessing two mating types, denominated *mat*+ and *mat–*. Sexual development in this fungus takes place inside multicellular perithecia. The fertilized ascogonial cells present in these structures possess nuclei of both mating types (illustrated by dots with different shading), which migrate into specialized hyphae emerging from ascogonia. These hyphae grow and bend producing hook-shaped cells called croziers. The two leading nuclei of croziers—possessing opposite mating type—undergo simultaneous mitoses (lines between nuclei depict spindles), and the formation of septa across the position formerly occupied by the spindles delimitates a dikaryotic upper cell from uninucleated lateral and basal cells. The upper dikaryotic cell undergoes karyogamy, differentiates into an ascus and enters meiosis, whereas the lateral and basal cells fuse and produce a new dikaryotic cell. The ascus elongates along meiotic prophase-I, and after ending meiosis a mitotic division yields eight haploid nuclei, which are enclosed by pairs into four ascospores. Ascospores grow inside the original ascus, increasing their volume about 10 times and differentiating a spherical head and a tail. Although formally heterothallic, *P. anserina* usually produces *mat*+*/mat–* dikaryotic ascospores, which after germination produce a heterokaryotic mycelium that is able to self-fertilize, this mating behavior is referred to as pseudo-homothallism. In addition, in a small percentage of asci (≈2%), one of the four binucleated ascospores produced is replaced by two small uninucleate ascospores, which usually posses opposite mating type and that upon germination yield homokaryotic mycelia.

Supplementary Figure 2 Molecular characterization of *P. anserina* Δ*fis1* **(A–C)** and Δ*dnm1* **(D–F)** mutants by PCR. **(A)** Schematics of the *FIS1* locus in the wild-type (top) and Δ*fis1* (bottom) strains. Red arrows indicate primers used to confirm the gene deletion (primer names, 1: Fis1-5ch-F, 2: Fis1-orf-R, 3: Fis1-orf-F, 4: Fis1-3ch-R, 5: nour-Rc, 6: nour-Fb). Green arrows indicate primers used to amplify the ORF flanking regions for the homologous recombination gene replacement (top), and the selectable marker (bottom). **(B)** Expected sizes of the PCR products amplified with different primer pairs, as indicated in **(A)**. **(C)** Confirmation of gene deletion by PCR. Δ*fis1* transformants 4 and 8 displayed the expected pattern for homologous recombination. **(D)** Schematics of the *DNM1* locus in the wild-type (top) and Δ*dnm1* (bottom) strains. Red and green arrows indicate the used primers as above (primer names, 1: dnm1-5ch-F, 2: dnm1-5ch-R, 3: dnm1-3ch-F, 4: dnm1-3ch-R, 5: hph-Rc, 6: hph-Fc). **(E)** Expected sizes of the PCR products amplified with different primer pairs, as indicated in **(D)**. **(F)** Confirmation of gene deletion by PCR. The Δ*dnm1* strain displayed the expected pattern for homologous recombination. M, DNA molecular weight marker; kb, kilobases.

Supplementary Figure 3 Growth phenotypes of *P. anserina* strains deficient for the peroxisome—mitochondrial fission machinery, or expressing IDHl-mCherry. **(A)** Growth curves of wild-type (WT), Δ*dnm1*, and Δ*fis1* strains on media containing dextrin (top) or oleic acid (bottom) as sole carbon sources. Values are mean ± SD of three independent experiments each with triplicates per strain. Statistical significant differences of Δ*dnm1* (blue) or Δ*fis1* (red) against the wild type by two- way Anova test are indicated (^*^*P* <0.05). **(B)** Colonial growth of WT, Δ*dnm1*, and Δ*fis1* strains at 72 h on dextrin (top)—and oleic acid (bottom plates)—containing media. **(C)** Colonial growth of WT and *IDHI::mCherry* strains (strains of both mating types are shown) at 48 h on dextrin (left) and oleic acid (center). The genotypes of the strains are indicated at right.

Supplementary Figure 4 Genetic complementation of Δ*dnm1* and Δ*fis1* strains. **(A,B)** Confocal microscopy analysis of mitochondria and peroxisomes in hyphae of WT, Δ*fis1*, and Δ*dnm1* strains, and of the same mutants complemented with a wild-type allele of the corresponding deleted gene (Δ*dnm1 DNM1*^+^ and Δ*fis1 FIS1*^+^, respectively). Peroxisomes were visualized with FOX2- GFP and mitochondria with MTS-mCherry **(A)** or MitoTracker Red **(B)**. BF, bright field. Scale bar, 5 μm. **(C)** Quantitation of the abnormal asci produced in WT, Δ*dnm1*, Δ*fis1*, Δ*dnm1 DNM1*^+^, and Δ*fis1 FIS1*^+^ sexual crosses (*n* ≥ 300 from three independent experiments. ^*^*P* <0.05 by unpaired Student's *t*-test).

Supplementary Figure 5 Analysis of Δ*dnm1* and Δ*fis1* mitochondria by MitoTracker Red staining. Confocal microscopy analysis of MitoTracker Red-labeled mitochondria in the apical and distal (~100 μm behind the hyphal tip) regions of WT **(A)**, Δ*fis1* **(B)**, and Δ*dnm1* **(C)** leading hyphae. In **(C)** mitochondria are compared to the localization of FOX2- GFP-labeled peroxisomes. BF, bright field. Scale bar, 5 μm.

Supplementary Figure 6 Effect of *DNM1* and *FIS1* deletion on peroxisome dynamics under different physiological conditions. Peroxisomes were visualized using FOX2-mCherry **(A)** or peroxisome-targeted GFP (GFP-PTS1) **(B)** in hyphae of the indicated genotypes growing on minimal medium containing dextrin or oleic acid as carbon sources, or grown in dextrin and exposed to low temperature (4°C) for 12 h. Scale bar, 5 μm.

Supplementary Figure 7 Peroxisome and mitochondrial arrangement in spermatia. Analysis of peroxisomes **(A,B)** and mitochondria **(C,D)** in WT, Δ*fis1*, and Δ*dnm1* spermatia. Epifluorescence microscopy analysis of peroxisomes **(A)** and mitochondria **(C)**. Peroxisomes were visualized with FOX2-mCherry (WT and Δ*fis1*) or with FOX2-GFP (Δ*dnm1*) and were pseudocolored red. Mitochondria were labeled with IDHl-mCherry. Arrows show elongated organelles. Arrowheads in **(A)** show beads-on-a-string peroxisomal chains, and in **(C)** large mitochondria. BF: bright field. Scale bar, 5 μm. Quantitation of spermatia containing peroxisomes **(B)** and mitochondria **(D)**. Values are mean ± SD of three independent experiments (*n* = 300, ^*^*P* <0.05 by unpaired Student's *t*-test).

Supplementary Table 1 *Podospora anserina* strains used in this research.

Supplementary Table 2 Oligonucleotide primers used in this research.
