# Supplementary material for: Distinct Contributions of the Peroxisome-Mitochondria Fission Machinery During Sexual Development of the Fungus Podospora anserina
Source: Front Microbiol. 2020 Apr 15;11:640. doi: 10.3389/fmicb.2020.00640 (PMC7175800; doi:10.3389/fmicb.2020.00640)
Supplement: Supplementary Figure 1 — Podospora anserina sexual development from the dikaryotic stage to ascospore formation (from left to right). P. anserina is an heterothallic ascomycete possessing two mating types, denominated mat+ and mat–. Sexual development in this fungus takes place inside multicellular perithecia. The fertilized ascogonial cells present in these structures possess nuclei of both mating types (illustrated by dots with different shading), which migrate into specialized hyphae emerging from ascogonia. These hyphae grow and bend producing hook-shaped cells called croziers. The two leading nuclei of croziers—possessing opposite mating type—undergo simultaneous mitoses (lines between nuclei depict spindles), and the formation of septa across the position formerly occupied by the spindles delimitates a dikaryotic upper cell from uninucleated lateral and basal cells. The upper dikaryotic cell undergoes karyogamy, differentiates into an ascus and enters meiosis, whereas the lateral and basal cells fuse and produce a new dikaryotic cell. The ascus elongates along meiotic prophase-I, and after ending meiosis a mitotic division yields eight haploid nuclei, which are enclosed by pairs into four ascospores. Ascospores grow inside the original ascus, increasing their volume about 10 times and differentiating a spherical head and a tail. Although formally heterothallic, P. anserina usually produces mat+/mat– dikaryotic ascospores, which after germination produce a heterokaryotic mycelium that is able to self-fertilize, this mating behavior is referred to as pseudo-homothallism. In addition, in a small percentage of asci (≈2%), one of the four binucleated ascospores produced is replaced by two small uninucleate ascospores, which usually posses opposite mating type and that upon germination yield homokaryotic mycelia. [file Data_Sheet_1.PDF]

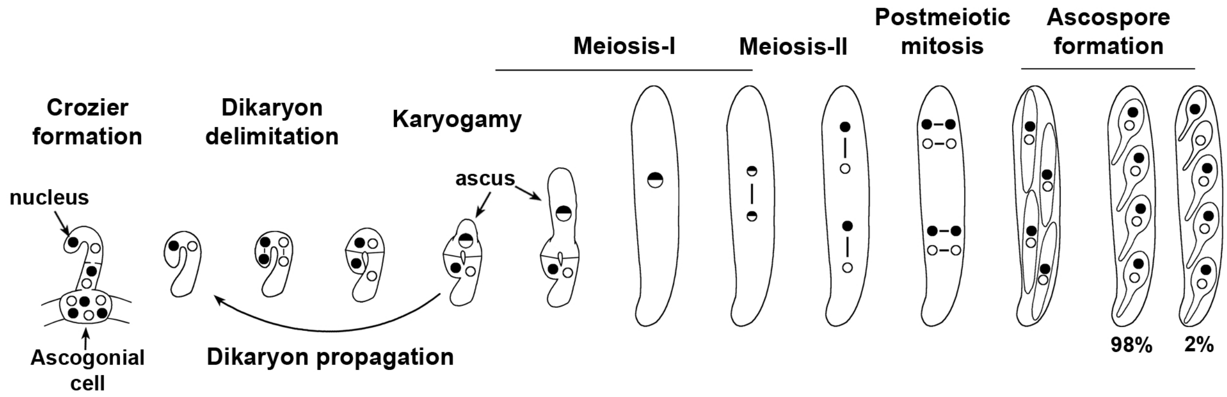

Supplementary Figure 1. *Podospora anserina* sexual development from the dikaryotic stage to ascospore formation (from left to right). *P. anserina* is an heterothallic ascomycete possessing two mating types, denominated *mat*+ and *mat*-. Sexual development in this fungus takes place inside multicellular perithecia. The fertilized ascogonial cells present in these structures possess nuclei of both mating types (illustrated by dots with different shading), which migrate into specialized hyphae emerging from ascogonia. These hyphae grow and bend producing hook-shaped cells called croziers. The two leading nuclei of croziers –possessing opposite mating type– undergo simultaneous mitoses (lines between nuclei depict spindles), and the formation of septa across the position formerly occupied by the spindles delimitates a dikaryotic upper cell from uninucleated lateral and basal cells. The upper dikaryotic cell undergoes karyogamy, differentiates into an ascus and enters meiosis, whereas the lateral and basal cells fuse and produce a new dikaryotic cell. The ascus elongates along meiotic prophase-I, and after ending meiosis a mitotic division yields eight haploid nuclei, which are enclosed by pairs into four ascospores. Ascospores grow inside the original ascus, increasing their volume about 10 times and differentiating a spherical head and a tail. Although formally heterothallic, *P. anserina* usually produces *mat*+/*mat*- dikaryotic ascospores, which after germination produce a heterokaryotic mycelium that is able to self-fertilize, this mating behavior is referred to as pseudo-homothallism. In addition, in a small percentage of asci (≈2%), one of the four binucleated ascospores produced is replaced by two small uninucleate ascospores, which usually posses opposite mating type and that upon germination yield homokaryotic mycelia.
