# Supplementary material for: Distinct Contributions of the Peroxisome-Mitochondria Fission Machinery During Sexual Development of the Fungus Podospora anserina
Source: Front Microbiol. 2020 Apr 15;11:640. doi: 10.3389/fmicb.2020.00640 (PMC7175800; doi:10.3389/fmicb.2020.00640)
Supplement: Supplementary Figure 2 — Molecular characterization of P. anserina Δfis1 (A–C) and Δdnm1 (D–F) mutants by PCR. (A) Schematics of the FIS1 locus in the wild-type (top) and Δfis1 (bottom) strains. Red arrows indicate primers used to confirm the gene deletion (primer names, 1: Fis1-5ch-F, 2: Fis1-orf-R, 3: Fis1-orf-F, 4: Fis1-3ch-R, 5: nour-Rc, 6: nour-Fb). Green arrows indicate primers used to amplify the ORF flanking regions for the homologous recombination gene replacement (top), and the selectable marker (bottom). (B) Expected sizes of the PCR products amplified with different primer pairs, as indicated in (A). (C) Confirmation of gene deletion by PCR. Δfis1 transformants 4 and 8 displayed the expected pattern for homologous recombination. (D) Schematics of the DNM1 locus in the wild-type (top) and Δdnm1 (bottom) strains. Red and green arrows indicate the used primers as above (primer names, 1: dnm1-5ch-F, 2: dnm1-5ch-R, 3: dnm1-3ch-F, 4: dnm1-3ch-R, 5: hph-Rc, 6: hph-Fc). (E) Expected sizes of the PCR products amplified with different primer pairs, as indicated in (D). (F) Confirmation of gene deletion by PCR. The Δdnm1 strain displayed the expected pattern for homologous recombination. M, DNA molecular weight marker; kb, kilobases. [file Data_Sheet_2.PDF]

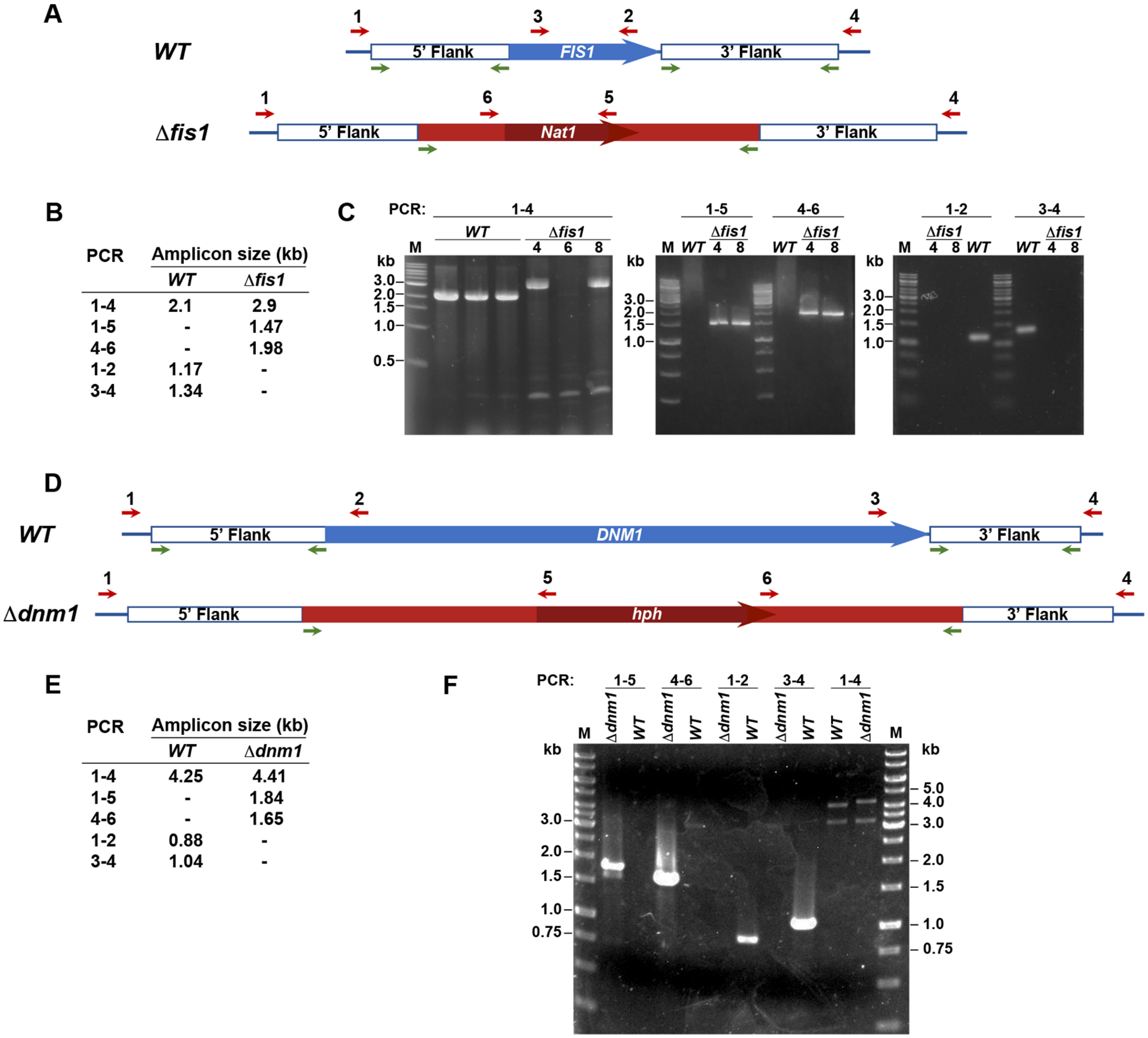

Supplementary Figure 2. Molecular characterization of *P. anserina Δfis1* (A-C) and *Δdnm1* (D-F) mutants by PCR. (A) Schematics of the *FIS1* locus in the wild-type (top) and *Δfis1* (bottom) strains. Red arrows indicate primers used to confirm the gene deletion (primer names, 1: Fis1-5ch-F, 2: Fis1-orf-R, 3: Fis1-orf-F, 4: Fis1-3ch-R, 5: nour-Rc, 6: nour-Fb). Green arrows indicate primers used to amplify the ORF flanking regions for the homologous recombination gene replacement (top), and the selectable marker (bottom). (B) Expected sizes of the PCR products amplified with different primer pairs, as indicated in (A). (C) Confirmation of gene deletion by PCR. *Δfis1* transformants 4 and 8 displayed the expected pattern for homologous recombination. (D) Schematics of the *DNM1* locus in the wild-type (top) and *Δdnm1* (bottom) strains. Red and green arrows indicate the used primers as above (primer names, 1: dnm1-5ch-F, 2: dnm1-5ch-R, 3: dnm1-3ch-F, 4: dnm1-3ch-R, 5: hph-Rc, 6: hph-Fc). (E) Expected sizes of the PCR products amplified with different primer pairs, as indicated in (D). (F) Confirmation of gene deletion by PCR. The *Δdnm1* strain displayed the expected pattern for homologous recombination. M, DNA molecular weight marker; kb, kilobases.
