# Supplementary material for: Distinct Contributions of the Peroxisome-Mitochondria Fission Machinery During Sexual Development of the Fungus Podospora anserina
Source: Front Microbiol. 2020 Apr 15;11:640. doi: 10.3389/fmicb.2020.00640 (PMC7175800; doi:10.3389/fmicb.2020.00640)
Supplement: Supplementary Figure 3 — Growth phenotypes of P. anserina strains deficient for the peroxisome—mitochondrial fission machinery, or expressing IDHl-mCherry. (A) Growth curves of wild-type (WT), Δdnm1, and Δfis1 strains on media containing dextrin (top) or oleic acid (bottom) as sole carbon sources. Values are mean ± SD of three independent experiments each with triplicates per strain. Statistical significant differences of Δdnm1 (blue) or Δfis1 (red) against the wild type by two- way Anova test are indicated (*P <0.05). (B) Colonial growth of WT, Δdnm1, and Δfis1 strains at 72 h on dextrin (top)—and oleic acid (bottom plates)—containing media. (C) Colonial growth of WT and IDHI::mCherry strains (strains of both mating types are shown) at 48 h on dextrin (left) and oleic acid (center). The genotypes of the strains are indicated at right. [file Data_Sheet_3.PDF]

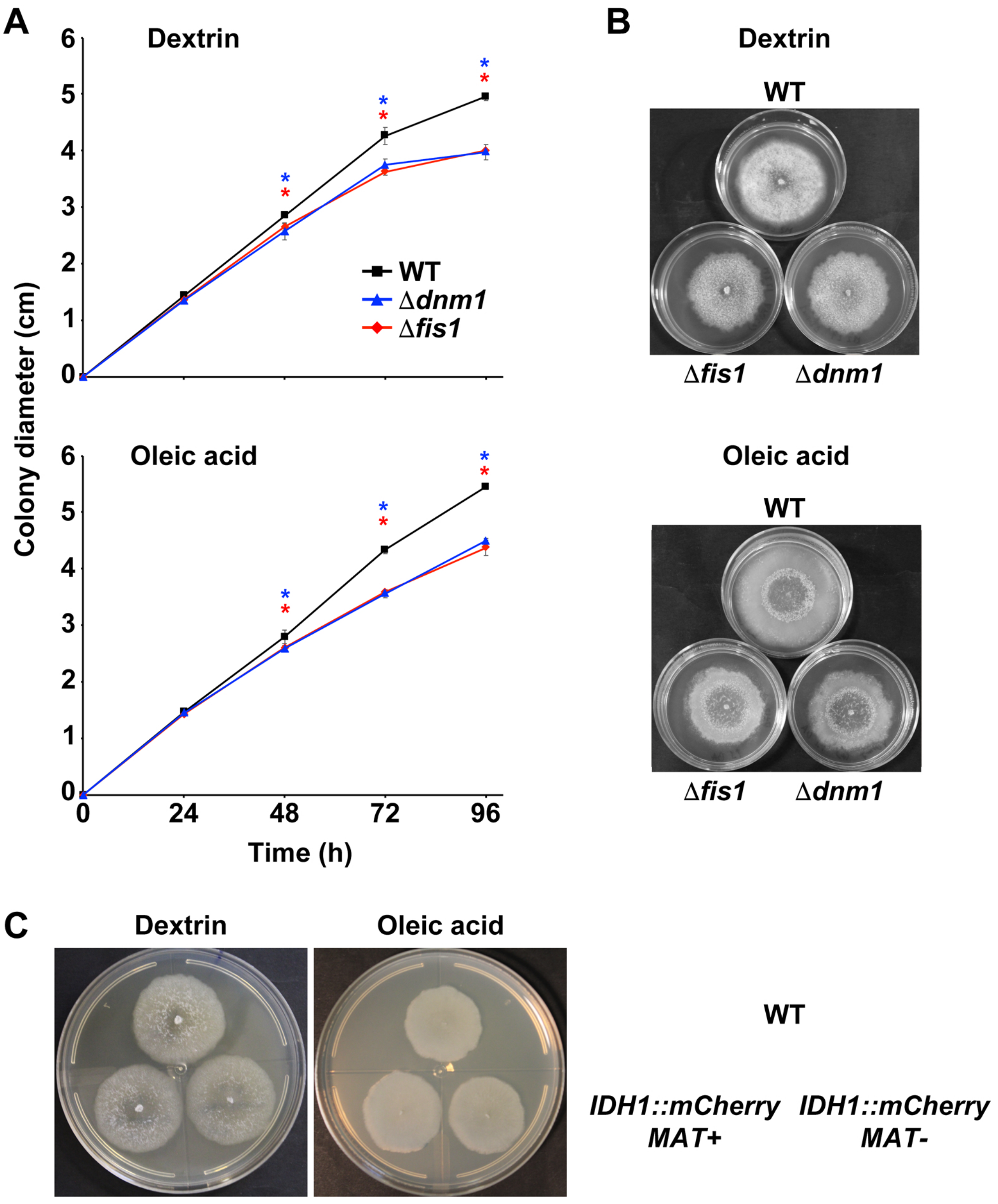

Supplementary Figure 3. Growth phenotypes of *P. anserina* strains deficient for the peroxisome-mitochondrial fission machinery, or expressing IDH1-mCherry. (A) Growth curves of wild-type (WT), *Δdnm1* and *Δfis1* strains on media containing dextrin (top) or oleic acid (bottom) as sole carbon sources. Values are mean ± SD of three independent experiments each with triplicates per strain. Statistical significant differences of *Δdnm1* (blue) or *Δfis1* (red) against the wild type by two-way Anova test are indicated (* $P<0.05$). (B) Colonial growth of WT, *Δdnm1* and *Δfis1* strains at 72 hours on dextrin (top)- and oleic acid (bottom plates)- containing media. (C) Colonial growth of WT and *IDH1::mCherry* strains (strains of both mating types are shown) at 48 hours on dextrin (left) and oleic acid (center). The genotypes of the strains are indicated at right.
