# Supplementary material for: Distinct Contributions of the Peroxisome-Mitochondria Fission Machinery During Sexual Development of the Fungus Podospora anserina
Source: Front Microbiol. 2020 Apr 15;11:640. doi: 10.3389/fmicb.2020.00640 (PMC7175800; doi:10.3389/fmicb.2020.00640)
Supplement: Supplementary Figure 4 — Genetic complementation of Δdnm1 and Δfis1 strains. (A,B) Confocal microscopy analysis of mitochondria and peroxisomes in hyphae of WT, Δfis1, and Δdnm1 strains, and of the same mutants complemented with a wild-type allele of the corresponding deleted gene (Δdnm1 DNM1+ and Δfis1 FIS1+, respectively). Peroxisomes were visualized with FOX2- GFP and mitochondria with MTS-mCherry (A) or MitoTracker Red (B). BF, bright field. Scale bar, 5 μm. (C) Quantitation of the abnormal asci produced in WT, Δdnm1, Δfis1, Δdnm1 DNM1+, and Δfis1 FIS1+ sexual crosses (n ≥ 300 from three independent experiments. *P <0.05 by unpaired Student's t-test). [file Data_Sheet_4.PDF]

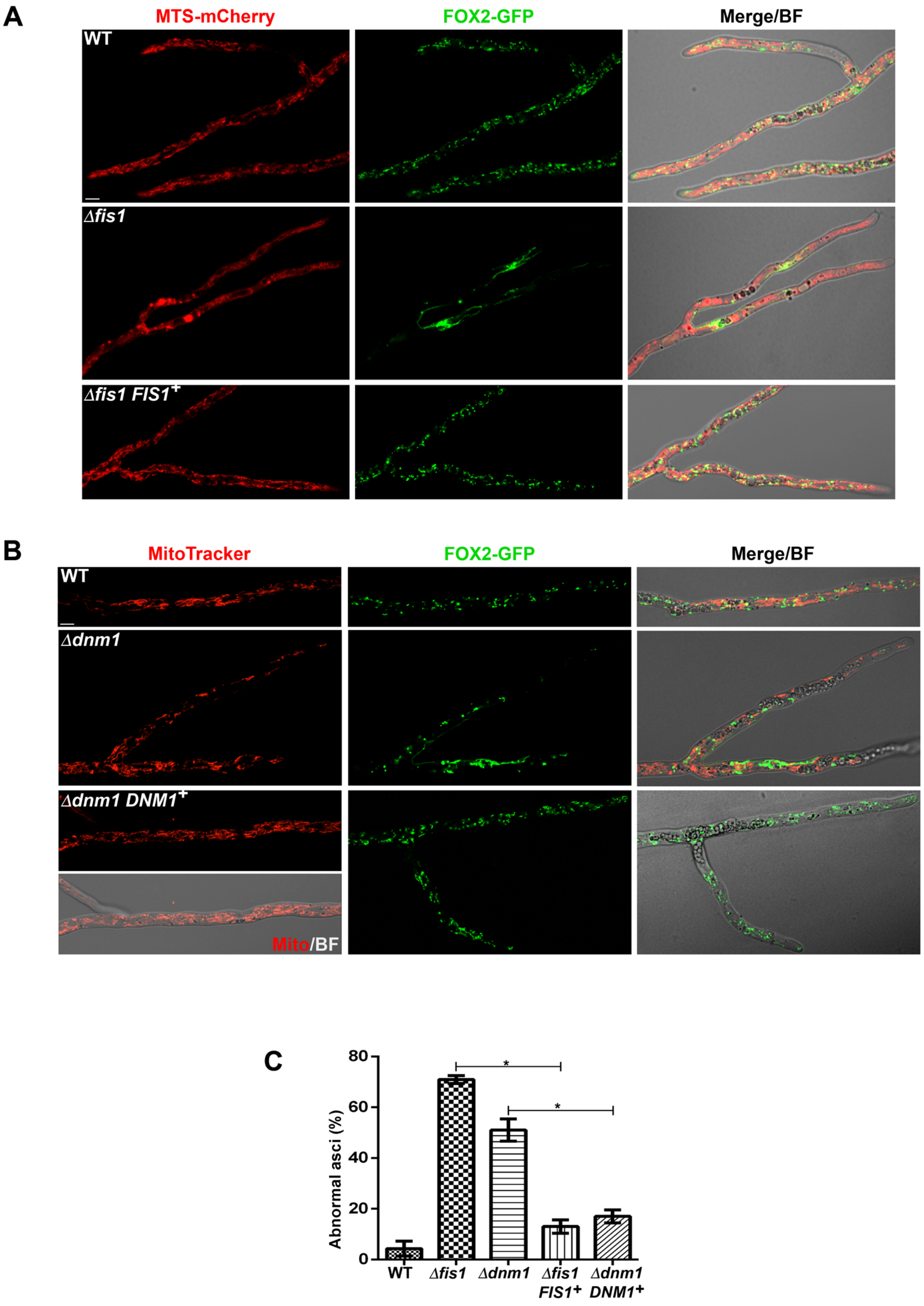

Supplementary Figure 4. Genetic complementation of *Δdnm1* and *Δfis1* strains. (A, B) Confocal microscopy analysis of mitochondria and peroxisomes in hyphae of WT, Δ*fis1* and Δ*dnm1* strains, and of the same mutants complemented with a wild-type allele of the corresponding deleted gene (Δ*dnm1 DNM1*$^{+}$ and Δ*fis1 FIS1*$^{+}$, respectively). Peroxisomes were visualized with FOX2-GFP and mitochondria with MTS-mCherry (A) or MitoTracker Red (B). BF: bright field. Scale bar, 5 μm. (C) Quantitation of the abnormal asci produced in WT, Δ*dnm1*, Δ*fis1*, Δ*dnm1 DNM1*$^{+}$ and Δ*fis1 FIS1*$^{+}$ sexual crosses ($n \geq 300$ from three independent experiments. *P<0.05 by unpaired Student's t test).
