# Supplementary material for: Distinct Contributions of the Peroxisome-Mitochondria Fission Machinery During Sexual Development of the Fungus Podospora anserina
Source: Front Microbiol. 2020 Apr 15;11:640. doi: 10.3389/fmicb.2020.00640 (PMC7175800; doi:10.3389/fmicb.2020.00640)
Supplement: Supplementary Figure 5 — Analysis of Δdnm1 and Δfis1 mitochondria by MitoTracker Red staining. Confocal microscopy analysis of MitoTracker Red-labeled mitochondria in the apical and distal (~100 μm behind the hyphal tip) regions of WT (A), Δfis1 (B), and Δdnm1 (C) leading hyphae. In (C) mitochondria are compared to the localization of FOX2- GFP-labeled peroxisomes. BF, bright field. Scale bar, 5 μm. [file Data_Sheet_5.PDF]

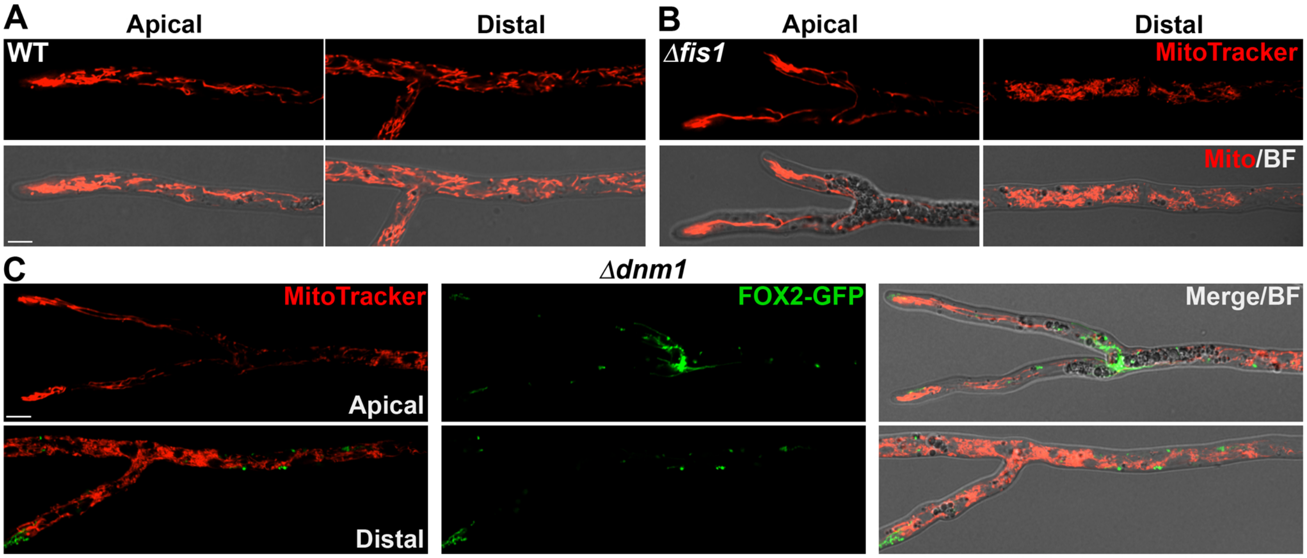

Supplementary Figure 5. Analysis of *Δdnm1* and *Δfis1* mitochondria by MitoTracker Red staining. Confocal microscopy analysis of MitoTracker Red-labeled mitochondria in the apical and distal (approximately 100 μm behind the hyphal tip) regions of WT (A), Δ*fis1* (B) and Δ*dnm1* (C) leading hyphae. In (C) mitochondria are compared to the localization of FOX2-GFP-labeled peroxisomes. BF: bright field. Scale bar, 5 μm.
