# Supplementary material for: Distinct Contributions of the Peroxisome-Mitochondria Fission Machinery During Sexual Development of the Fungus Podospora anserina
Source: Front Microbiol. 2020 Apr 15;11:640. doi: 10.3389/fmicb.2020.00640 (PMC7175800; doi:10.3389/fmicb.2020.00640)
Supplement: Supplementary Figure 6 — Effect of DNM1 and FIS1 deletion on peroxisome dynamics under different physiological conditions. Peroxisomes were visualized using FOX2-mCherry (A) or peroxisome-targeted GFP (GFP-PTS1) (B) in hyphae of the indicated genotypes growing on minimal medium containing dextrin or oleic acid as carbon sources, or grown in dextrin and exposed to low temperature (4°C) for 12 h. Scale bar, 5 μm. [file Data_Sheet_6.PDF]

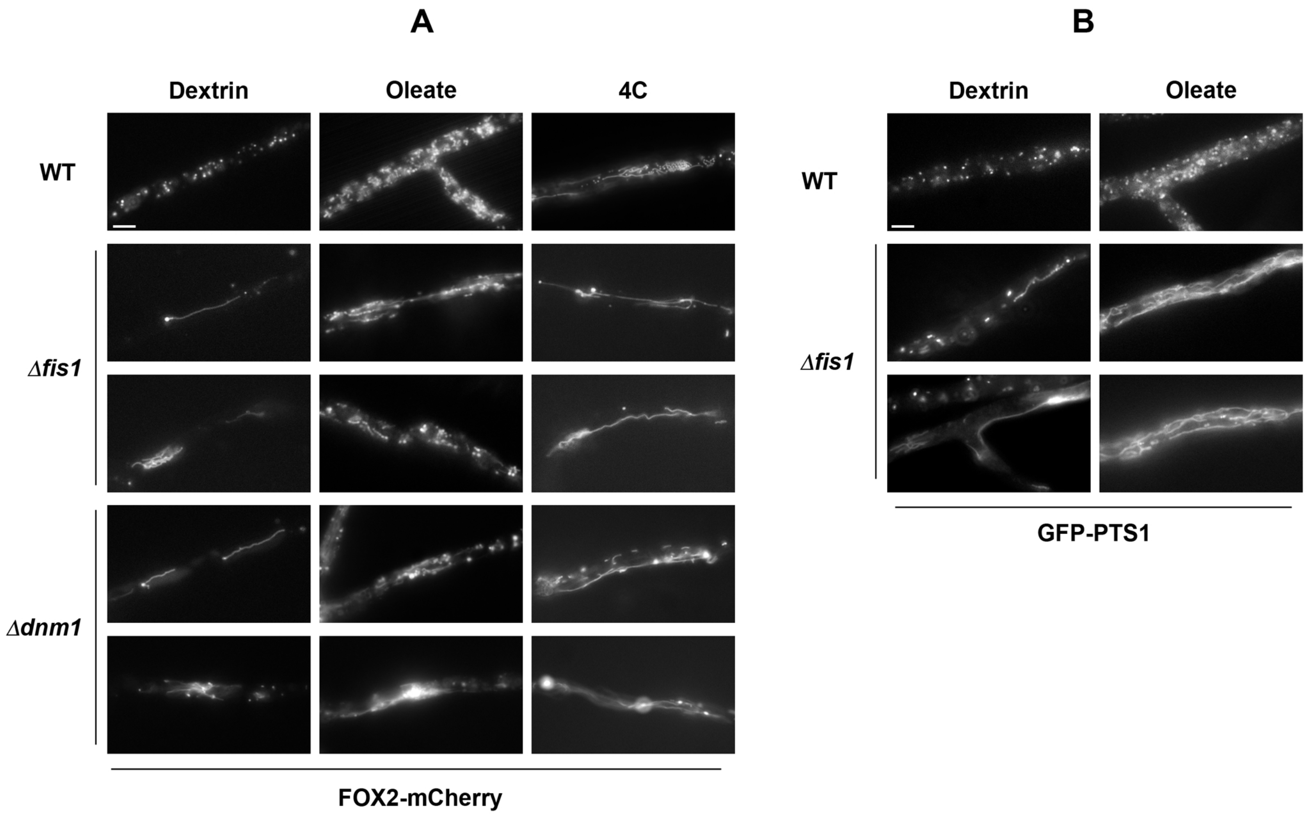

Supplementary Figure 6. Effect of *DNM1* and *FIS1* deletion on peroxisome dynamics under different physiological conditions. Peroxisomes were visualized using FOX2-mCherry (A) or peroxisome-targeted GFP (GFP-PTS1) (B) in hyphae of the indicated genotypes growing on minimal medium containing dextrin or oleic acid as carbon sources, or grown in dextrin and exposed to low temperature (4 C) for 12h. Scale bar, 5μm.
