# Supplementary material for: Distinct Contributions of the Peroxisome-Mitochondria Fission Machinery During Sexual Development of the Fungus Podospora anserina
Source: Front Microbiol. 2020 Apr 15;11:640. doi: 10.3389/fmicb.2020.00640 (PMC7175800; doi:10.3389/fmicb.2020.00640)
Supplement: Supplementary Figure 7 — Peroxisome and mitochondrial arrangement in spermatia. Analysis of peroxisomes (A,B) and mitochondria (C,D) in WT, Δfis1, and Δdnm1 spermatia. Epifluorescence microscopy analysis of peroxisomes (A) and mitochondria (C). Peroxisomes were visualized with FOX2-mCherry (WT and Δfis1) or with FOX2-GFP (Δdnm1) and were pseudocolored red. Mitochondria were labeled with IDHl-mCherry. Arrows show elongated organelles. Arrowheads in (A) show beads-on-a-string peroxisomal chains, and in (C) large mitochondria. BF: bright field. Scale bar, 5 μm. Quantitation of spermatia containing peroxisomes (B) and mitochondria (D). Values are mean ± SD of three independent experiments (n = 300, *P <0.05 by unpaired Student's t-test). [file Data_Sheet_7.pdf]

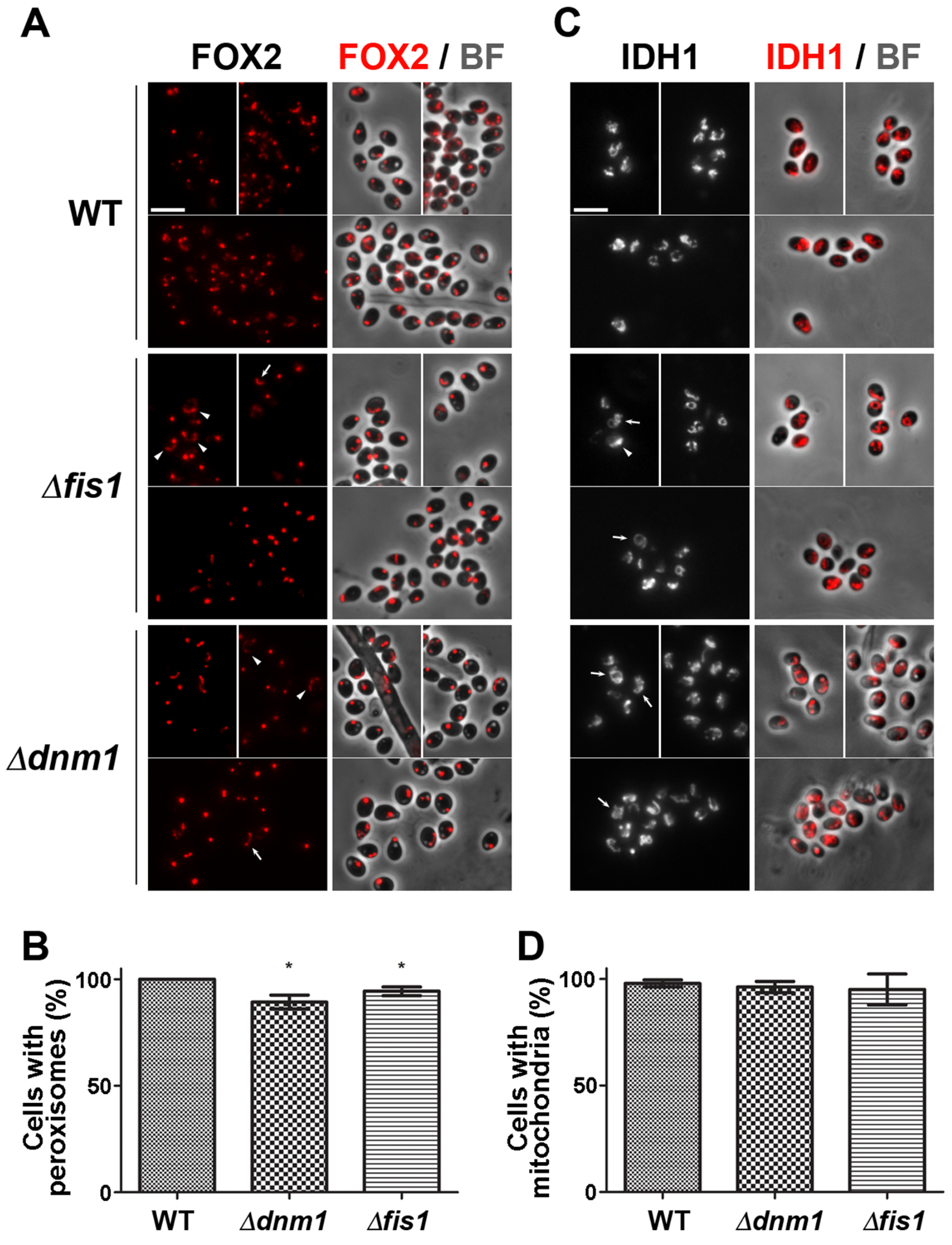

Supplementary Figure 7. Peroxisome and mitochondrial arrangement in spermatia. Analysis of peroxisomes (A-B) and mitochondria (C-D) in WT, *Δfis1* and *Δdnm1* spermatia. Epifluorescence microscopy analysis of peroxisomes (A) and mitochondria (C). Peroxisomes were visualized with FOX2-mCherry (WT and *Δfis1*) or with FOX2-GFP (*Δdnm1*) and were pseudocolored red. Mitochondria were labeled with IDH1-mCherry. Arrows show elongated organelles. Arrowheads in (A) show beads-on-a-string peroxisomal chains, and in (C) large mitochondria. BF: bright field. Scale bar, 5μm. Quantitation of spermatia containing peroxisomes (B) and mitochondria (D). Values are mean ± SD of three independent experiments (n=300, *P<0.05 by unpaired Student's t test).
