# Supplementary material for: Distinct Contributions of the Peroxisome-Mitochondria Fission Machinery During Sexual Development of the Fungus Podospora anserina
Source: Front Microbiol. 2020 Apr 15;11:640. doi: 10.3389/fmicb.2020.00640 (PMC7175800; doi:10.3389/fmicb.2020.00640)
Supplement: Supplementary Table 1 — Podospora anserina strains used in this research. [file Table_1.pdf]

Supplementary Table 1. *Podospora anserina* strains used in this research.

Strain	Genotype	Source
S+ (*Wt*)	*mat+*	Rizet, 1952
S- (*Wt*)	*mat-*	Rizet, 1952
ku70	*Δku70::geneticinR mat+*	R. Debuchy*
GFP-PTS1	*GFP-SKL::ble(EC) mat+*	Ruprich-Robert, at al, 2002
FS19	*FOX2::mCherry mat-*	Suaste-Olmos, et al, 2018
FS20	*FOX2::mCherry mat+*	Suaste-Olmos, et al, 2018
FS23	*FOX2::GFP mat+*	This study
FS24	*FOX2::GFP mat-*	This study
FS130	*IDH1::mCherry mat+*	This study
FS131	*IDH1::mCherry mat-*	This study
FS134	*ATP9-7::mCherry(EC) mat+*	This study
LP206	*FOX2::GFP ATP9-7::mCherry(EC) mat-*	This study
HT07	*Δfis1::nat1 4D mat+*	This study
HT08	*Δfis1::nat1 4D mat-*	This study
HT10	*Δfis1::nat1 8B mat+*	This study
HT11	*Δfis1::nat1 GFP-SKL::ble(EC) mat-*	This study
HT12	*Δfis1::nat1 GFP-SKL::ble(EC) mat+*	This study
HT17	*Δfis1::nat1 FOX2::mCherry GFP-SKL::ble(EC) mat-*	This study
HT18	*Δfis1::nat1 FOX2::mCherry GFP-SKL::ble(EC) mat+*	This study
HT33	*Δfis1::nat1 FOX2::mCherry mat+*	This study
HT34	*Δfis1::nat1 FOX2::mCherry mat-*	This study
LP207	*Δfis1::nat1 FOX2::GFP ATP9-7::mCherry(EC) mat+*	This study

HT35	*Δfis1::nat1 FOX2::GFP ATP9-7::mCherry(EC) FIS1+(EC)-1 mat+*	This study
HT36	*Δfis1::nat1 FOX2::GFP ATP9-7::mCherry(EC) FIS1+(EC)-6 mat+*	This study
FS132	*Δfis1::nat1 FOX2::GFP mat+*	This study
FS133	*Δfis1::nat1 FOX2::GFP mat-*	This study
RN1	*FOX2::GFP IDH1::mCherry mat+*	This study
RN5	*FOX2::GFP IDH1::mCherry mat-*	This study
RN10	*Δdnm1::hph FOX2::GFP mat+*	This study
RN11	*Δdnm1::hph FOX2::GFP mat-*	This study
RN13	*Δdnm1::hph IDH1::mCherry mat-*	This study
RN14	*Δdnm1::hph mat+*	This study
RN15	*Δdnm1::hph mat-*	This study
RN16	*Δdnm1::hph FOX2::GFP IDH1::mCherry mat+*	This study
RN17	*Δdnm1::hph FOX2::GFP DNM1+(EC)-C1 mat+*	This study
RN23	*Δdnm1::hph FOX2::GFP DNM1+(EC)-C5 mat+*	This study
RN18	*Δdnm1::hph FOX2::GFP DNM1+(EC)-C9 mat+*	This study
RN19	*Δdnm1::hph FOX2::GFP DNM1+(EC)-C11 mat+*	This study
RN20	*Δdnm1::hph FOX2::GFP DNM1+(EC)-C12 mat+*	This study
RN21	*Δdnm1::hph FOX2::GFP DNM1+(EC)-C13 mat+*	This study
RN22	*Δdnm1::hph FOX2::GFP DNM1+(EC)-C14 mat+*	This study
RN24	*Δdnm1::hph FOX2::GFP DNM1+(EC)-C21 mat+*	This study
RN25	*Δdnm1::hph FOX2::GFP DNM1+(EC)-C31 mat+*	This study

*Kindly provided by Dr. Robert Debuchy, Institute for Integrative Biology of the Cell, CEA, CNRS, Univ. Paris-Sud, Université Paris-Saclay, France.

References

Suaste-Olmos, F., Zirion-Martinez, C., Takano-Rojas, H., and Peraza-Reyes, L. (2018). Meiotic development initiation in the fungus *Podospora anserina* requires the peroxisome receptor export machinery*. Biochim Biophys Acta 1865(4)*, *572-586.*

Rizet G. (1952). Les phénomènes de barrage chez *Podospora anserina*. I. Analyse génétique des barrages entre souches *S* and *s*. *Rev Cytol Biol Veg* 13, 51-92.

Ruprich-Robert, G., Berteaux-Lecellier, V., Zickler, D., Panvier-Adoutte, A., and Picard, M. (2002). Identification of six loci in which mutations partially restore peroxisome biogenesis and/or alleviate the metabolic defect of pex2 mutants in *Podospora*. *Genetics* 161(3), 1089-1099.
