# Supplementary material for: Distinct Contributions of the Peroxisome-Mitochondria Fission Machinery During Sexual Development of the Fungus Podospora anserina
Source: Front Microbiol. 2020 Apr 15;11:640. doi: 10.3389/fmicb.2020.00640 (PMC7175800; doi:10.3389/fmicb.2020.00640)
Supplement: Supplementary Table 2 — Oligonucleotide primers used in this research. [file Table_2.pdf]

Supplementary Table 2. Oligonucleotide primers used in this research.

Primer name	Primer sequence 5'>3'
Fis1-5F	CTGCTTTTTGAAGGGGGCAGTGGAG
Fis1-5R	GAAGCATTTATCAGGGTTGTCATGGTGGGCAAAAC
Fis1-3F	GTATTCAACATTTCCGTGGATAACGACAGCAAGGGC
Fis1-3R	CAGCAGCAACTTGTCCTGATTGCCA
Fis-NourF	GTTTTGCCCACCATGACAACCCTGATAAATGCTTC
Fis-NourR	GCCCTTGCTGTCGTTATCCACGGAAATGTTGAATAC
Fis1-5ch-F	GATTGTGTGGCGGATTAG
Fis1-3ch-R	TATCATCCTTAGCGCGAA
Fis1-orf-F	CGAGAACATGAAGTGCTT
Fis1-orf-R	TTCGAAGCTTGAAGGTTG
nour-Fb	CGACATCTCATCTTCCTG
nour-Rc	GTTGACGTTGGTGACCT
dnm1-5F	CCTAAAGTTAGGTACTGGCAGCCAC
dnm1-5R	ATGATTCTTCTCGCTTCCGCAGGGCCACAAGAAACT
dnm1-3F	CCTTCTTTCTAGCTAGAGGGGCTAGTTGGGCAAGTGG
dnm1-3R	GTGTCTGGTGGGAGAGAATAGAACC
dnm-hphF	AGTTTCTTGTGGCCCTGCGGAAGCGAGAAGAATCAT
dnm-hphR	CCACTTGCCCAACTAGCCCCTCTAGCTAGAAAGAAGG
dnm1-5chF	GGTAACTCAACTGGGTGCC
dnm1-5chR	TCTGGGGCAGATCGAGAG
dnm1-3chF	CACTGCAAGGACGTGGTT
dnm1-3chR	AGAAGATGGCGTCGTTGC
hph-Rc	GCGGTGAGTTCAGGCTTT

hph-Fc	CGCCGATAGTGGAAACCG
Fox2-F	TTCCCCCATTCGATACCGAGTTCCC
lkt-fox2	TAAACCAGCACCGTCACCAAGCAACTCCGCCGCGGC
fox-lkt	GCCGCGGCGGAGTTGCTTGGTGACGGTGCTGGTTTA
fox-hph	CTAACATCATCACTTGAATTATTCCTTTGCCCTCGGA
fox2-3F	TCCGAGGGCAAAGGAATAATTCAAGTGATGATGTTAG
fox2-3R	AAATGTGCGACTGGGTAGGTAGTCT
Idh1-F	CATCATCCGTGAGAATACCGAGGGC
lkt-idh1	TAAACCAGCACCGTCACCCAACGAGTTCTCCATGGAG
idh-lkt	CTCCATGGAGAACTCGTTGGGTGACGGTGCTGGTTTA
idh-hph	CGTGTGTGGTTGGGTATCTTATTCCTTTGCCCTCGGA
idh1-3F	TCCGAGGGCAAAGGAATAAGATACCCAACCACACACG
idh1-3R	AAGAAACCCCTTGGCACTTCTAGCG
pgpd-F	AATTCTTCTAGAATCCCTTC
pgpd-R	GCTTGCTAGAGGCGTTCATGGTTGCTGTTGGTGAAG
mts-F	CTTCACCAACAGCAACCATGAACGCCTCTAGCAAGC
mts-R	CTCCTCGCCCTTGCTCACGGCGGCCGTGGCAGTCTC
mts-gfp-F	GAGACTGCCACGGCCGCCGTGAGCAAGGGCGAGGAG
gfp-R	CACCAAAACCATCATGTCTACTTGTACAGCTCGTCCA
